# Multi-objective deep deterministic policy gradient for cobot trajectory planning in automotive spot welding

**DOI:** 10.3389/frobt.2026.1939068

**Published:** 2026-10-20

**Authors:** Batyrkhan Omarov

**Affiliations:** 1 Laboratory of Mechanics of Robots and Manipulators, Joldasbekov Institute of Mechanics and Engineering, Almaty, Kazakhstan; 2 Department of Mathematical and Computer Modeling, International Information Technology University, Almaty, Kazakhstan; 3 School of Digital Technologies, Narxoz University, Almaty, Kazakhstan

**Keywords:** collaborative robot, DDPG, industrial robotics, multi-objective optimization, reinforcement learning, spot welding, trajectory planning

## Abstract

**Introduction:**

Autonomous trajectory planning is a fundamental challenge in collaborative robotic spot welding because positioning accuracy, motion smoothness, operational safety, energy use, and production efficiency must be optimized simultaneously under industrial constraints.

**Methods:**

This paper presents a Pareto-Guided Multi-Objective Deep Deterministic Policy Gradient (PMO-DDPG) framework for autonomous cobot trajectory planning in automotive spot welding. The framework combines a deterministic actor with four objective-specific critics for accuracy, smoothness, safety, and productivity, together with phase-dependent preferences for the approach, alignment, welding, and retraction stages. The principal novelty is a two-level multi-objective design that preserves six normalized physical reward components, maps them to four specialized critics for phase-dependent actor updates, and uses a non-dominated policy archive for Pareto-guided selection while retaining actor-only online inference. Simulation and industrial experiments assessed convergence, trajectory quality, collision avoidance, energy consumption, cycle time, success rate, and latency.

**Results:**

Relative to the Soft Actor-Critic (SAC) baseline, PMO-DDPG reduced trajectory error by 66.4%, collision rate by 82.1%, energy consumption by 15.5%, cycle time by 22.0%, and inference latency by 22.7%, while increasing the success rate by 7.5 percentage points. The proposed method achieved 1.08 mm trajectory error, a 1.2% collision rate, 3.65 kJ energy consumption, a 3.87 s cycle time, a 94.6% success rate, and 21.5 ms inference time. Pareto-front analysis produced non-dominated operating points for different manufacturing priorities, and 100 industrial trials confirmed sub-millimeter positioning and repeatable vision-guided welding.

**Discussion:**

Within the evaluated simulation and industrial test settings, and without a formal significance test, these descriptive results indicate that PMO-DDPG is a promising accurate, safe, and efficient trajectory-planning approach.

## 1 Introduction

The automotive industry continues to pursue highly automated manufacturing systems capable of improving productivity, consistency, and operational safety while reducing production costs and human intervention. Among various manufacturing processes, resistance spot welding remains one of the most extensively employed joining technologies for vehicle body assembly because thousands of welds are required to fabricate a single body-in-white structure (Wang, 2025). Although industrial robots have significantly increased manufacturing throughput, trajectory generation for spot-welding operations remains a challenging problem due to the simultaneous need to satisfy multiple conflicting objectives, including positioning accuracy, motion smoothness, collision avoidance, cycle time, and energy efficiency (Das, 2026). Conventional trajectory planning methods, such as linear interpolation, spline-based approaches, sampling-based planners, and optimization-based controllers, generally optimize one or two objectives and often require carefully tuned mathematical models that become increasingly difficult to maintain in dynamic industrial environments. Recent advances in intelligent robotics have therefore shifted attention toward data-driven decision-making methods capable of continuously adapting robot behavior according to changing workspace conditions and production requirements (Tang et al., 2025).

Deep reinforcement learning (DRL) has recently emerged as an effective paradigm for solving sequential decision-making problems in robotics because it directly learns control policies through interactions with the environment rather than relying on explicit analytical models. Policy-gradient and actor-critic algorithms, including Deep Deterministic Policy Gradient (DDPG), Twin Delayed DDPG (TD3), Proximal Policy Optimization (PPO), and Soft Actor-Critic (SAC), have demonstrated considerable success in continuous robotic control tasks such as manipulation, mobile navigation, obstacle avoidance, and autonomous trajectory planning (Momynkulov et al., 2026). Their ability to generate continuous actions makes them particularly suitable for industrial manipulators that require precise end-effector positioning and smooth motion. Nevertheless, practical deployment of these methods in manufacturing environments remains challenging because conventional reinforcement learning algorithms typically optimize a single scalar reward, forcing multiple performance criteria into a fixed weighted objective whose parameters must be manually determined. Such formulations frequently lead to unstable training, conflicting optimization objectives, and policies that perform well in one criterion while sacrificing others (Hirtz et al., 2025).

Recent studies have consequently investigated multi-objective reinforcement learning to enable simultaneous optimization of several task-specific objectives (Zhang Z. et al., 2026; Ibarayev et al., 2024). Adaptive reward weighting, multiple critic architectures, and Pareto-based policy evaluation have demonstrated promising capabilities for balancing competing goals while improving robustness and generalization across complex robotic applications. Likewise, recent reviews have emphasized that future industrial reinforcement learning systems should incorporate adaptive objective balancing, sample-efficient learning, and real-world validation to facilitate reliable deployment in manufacturing scenarios (Hillali et al., 2025). Furthermore, advances in deep reinforcement learning for robotic path planning indicate that0 trajectory quality depends not only on collision-free navigation but also on smoothness, computational efficiency, and robustness under dynamic constraints (Hillali et al., 2025). Similarly, practical investigations have shown that carefully designed reward shaping and continuous-control algorithms substantially improve trajectory tracking accuracy and motion stability for robotic manipulators operating in uncertain environments (Fareh et al., 2025).

Motivated by these challenges, this paper proposes Pareto-Guided Multi-Objective Deep Deterministic Policy Gradient (PMO-DDPG) for autonomous cobot trajectory planning in automotive spot welding. The proposed framework extends conventional DDPG by introducing four objective-specific critic networks dedicated to trajectory accuracy, motion smoothness, operational safety, and production efficiency. An adaptive Pareto preference module dynamically adjusts objective priorities according to the current welding phase, allowing the policy to emphasize different operational requirements during approach, alignment, welding, and retraction. Instead of relying on a fixed scalar reward, the proposed method combines objective-specific evaluations through Pareto-guided aggregation, enabling the learning process to identify balanced non-dominated policies for complex industrial tasks (Han et al., 2026). The effectiveness of the proposed framework is validated through comprehensive simulation experiments, comparisons with state-of-the-art reinforcement learning methods, vision-guided target localization, and real industrial spot-welding case studies, demonstrating its capability to generate accurate, smooth, safe, and efficient trajectories suitable for intelligent automotive manufacturing environments.

The principal contributions of this study are summarized as follows:

1. A multi-objective DDPG architecture is developed with one deterministic actor and four objective-specific critics, reducing value-function interference among accuracy, smoothness, safety, and productivity.
2. A transparent six-component final reward is introduced with phase-dependent weights, an adaptive performance-deficit update, and an explicit mapping from component preferences to the four critic gradients.
3. Pareto guidance is integrated through non-dominated checkpoint retention, archive-derived reference objectives, and ideal-point policy selection, enabling manufacturing priorities to change without retraining the online actor.
4. The method is evaluated through controlled comparisons, Pareto analysis, domain-randomized simulation, vision-guided localization, and repeated real industrial spot-welding experiments.

## 2 Related works

Trajectory planning has long been recognized as one of the fundamental problems in industrial robotics because it directly influences positioning accuracy, execution time, energy consumption, and operational safety. Classical methods, including linear interpolation, polynomial interpolation, cubic splines, B-splines, and Bézier curves, remain widely used owing to their computational efficiency and smooth path generation capabilities (Sun et al., 2023; Chaudhuri, 2024; An et al., 2025). Graph-search algorithms, such as A* and Dijkstra-based planners, have also been extensively adopted for collision-free navigation in structured environments, while sampling-based methods improve exploration in high-dimensional configuration spaces (Pogaçe and Karras, 2022). More recently, model predictive control (MPC) has gained popularity because it explicitly incorporates system dynamics and operational constraints during online optimization (Harbi et al., 2023). Despite these advantages, conventional planners generally optimize predefined cost functions and require accurate mathematical models, limiting their adaptability to dynamic industrial environments containing moving obstacles, uncertain robot dynamics, and continuously changing manufacturing conditions (Chen et al., 2023; Raj and Kos, 2024).

Deep reinforcement learning has significantly advanced continuous robotic control by enabling manipulators to learn optimal policies directly from interactions with their environments. Among existing methods, Deep Deterministic Policy Gradient (DDPG) introduced deterministic actor–critic learning for continuous action spaces, while Twin Delayed DDPG (TD3) addressed value overestimation through delayed policy updates and twin critic networks (Deshpande et al., 2024; Hazem, 2026). Proximal Policy Optimization (PPO) provides stable policy optimization using clipped surrogate objectives and has demonstrated strong performance in robotic manipulation tasks (Boudlal et al., 2024). Soft Actor-Critic (SAC) further improves exploration by maximizing policy entropy while maintaining sample efficiency (Zhang D et al., 2026). Modern actor–critic frameworks additionally employ replay buffers, target networks, and stochastic exploration strategies to stabilize learning and improve convergence in continuous control problems (Wen et al., 2025). Nevertheless, most existing reinforcement learning methods rely on single-objective reward formulations that inadequately capture the multiple competing objectives encountered in industrial robotic applications.

Beyond direct robot control, deep reinforcement learning has become a general optimization framework for sequential problems in which decisions alter future costs and constraints. Value-based methods are effective when the optimization variables are discrete, as in routing, sequencing, and scheduling, whereas policy-gradient methods directly optimize continuous decision variables. Actor-critic algorithms combine both ideas: the critic estimates long-term objective values and the actor follows their gradients to improve a continuous policy. DDPG provides sample-efficient deterministic control, TD3 reduces overestimation with twin critics and delayed updates, PPO constrains policy changes through a clipped objective, and SAC adds entropy maximization to improve exploration. Across manufacturing, energy management, navigation, and robotic manipulation, the optimization objective is encoded through rewards for makespan, energy, tracking, collision risk, resource utilization, or quality (Tang et al., 2025; Hirtz et al., 2025; Xiupeng et al., 2023; Fareh et al., 2025).

Multi-objective DRL extends this formulation by retaining an objective vector instead of collapsing all criteria prematurely. Fixed scalarization is simple but sensitive to reward scale and cannot represent changing priorities. Decomposition and multi-critic approaches learn objective-specific returns, while preference conditioning and Pareto archives expose several non-dominated operating policies rather than one compromise. The present study adopts this decomposition because spot welding is continuous, constrained, phase dependent, and governed by objectives with different physical units and temporal behavior (Felten et al., 2024; He et al., 2026).

Model predictive control and reinforcement learning have also been applied beyond industrial manipulator trajectory planning. MPC has been used for constrained power-electronic control, where switching and current limits must be enforced online, and for risk-aware aerial-vehicle motion planning under dynamic obstacles (Harbi et al., 2023; Chen et al., 2023). Reinforcement learning has addressed unknown-environment mobile-robot navigation, autonomous-vehicle collision avoidance, and continuous manipulator tracking without requiring a fully accurate analytical model (Raj and Kos, 2024; Muzahid et al., 2022; Fareh et al., 2025). These applications clarify the complementary trade-off: MPC offers explicit finite-horizon constraint handling but repeatedly solves an online optimization problem, whereas RL transfers the main optimization cost to training and executes a learned policy rapidly. The proposed framework uses learned actor inference and retains an explicit downstream constraint-checking layer for industrial safety.

Multi-objective reinforcement learning (MORL) extends conventional reinforcement learning by simultaneously optimizing several conflicting objectives instead of maximizing a single scalar reward. Early approaches primarily employed scalarization through fixed weighted sums, requiring manual selection of objective weights that remain constant throughout training (Felten et al., 2024; Ran et al., 2026). Dynamic weighting strategies were later introduced to adjust objective importance according to environmental conditions or task phases, improving flexibility in complex decision-making scenarios (Digkoglou and Papathanasiou, 2025). More recent studies have explored multi-critic architectures, where independent critics estimate objective-specific value functions before aggregation into a unified policy update (Yang et al., 2025). Pareto dominance has become an increasingly important concept because it identifies non-dominated policies representing optimal trade-offs among conflicting objectives, enabling policy-set generation instead of a single operating solution (Li et al., 2026; Alegre et al., 2026). However, relatively few studies integrate adaptive objective weighting, Pareto-guided policy selection, and continuous robotic trajectory planning within a unified reinforcement learning framework.

Automotive spot welding presents unique challenges that distinguish it from many other robotic manipulation tasks. Accurate robot positioning and precise welding-gun orientation are essential to ensure weld quality while avoiding excessive electrode wear and structural deformation (Ma et al., 2025). Modern production lines also impose strict cycle-time constraints that require robots to minimize unnecessary movements without compromising positioning precision or operational safety (Asadi et al., 2025). The body-in-white assembly environment is characterized by dense metallic structures, narrow access regions, and multiple collision hazards that significantly complicate trajectory planning (Aminzadeh et al., 2026; Konstantinidis et al., 2023). In addition, reliable force control and vision-based target localization have become increasingly important for compensating manufacturing tolerances and ensuring consistent weld quality under varying operating conditions (Ma et al., 2025; Song et al., 2025). Despite considerable progress, transferring reinforcement learning algorithms from simulation to industrial automotive spot-welding systems remains challenging because of safety requirements, domain discrepancies, and limited opportunities for online exploration.

Robot calibration is closely related to trajectory execution because absolute kinematic error can propagate into the state estimate and welding-target pose. Calibration research has progressed from systematic classifications of geometric, non-geometric, and measurement procedures to open-dataset learning systems, regularized neural calibration, and multi-planar constraint formulations (Li et al., 2021; Li et al., 2022; Li et al., 2023; Chen et al., 2024). A recent data-driven survey further organizes calibration models, optimization algorithms, datasets, metrics, and industrial applications (Chen et al., 2025). These methods do not replace trajectory planning; rather, they improve the accuracy of the robot model on which perception, state construction, and execution depend. In the present framework, vision-based target-offset compensation complements calibration by correcting residual workpiece and tool-center-point deviations before the PMO-DDPG action is executed.

The reviewed literature demonstrates substantial progress in robotic trajectory planning, continuous-control reinforcement learning, and multi-objective optimization. Nevertheless, important research gaps remain for intelligent automotive spot welding. Existing reinforcement learning methods typically employ single or shared critics that cannot independently optimize trajectory accuracy, motion smoothness, operational safety, and production efficiency (Muzahid et al., 2022; Yang and Liu, 2024). Similarly, most multi-objective reinforcement learning studies rely on static scalarization or generic Pareto optimization without considering adaptive phase-dependent objective weighting required during different welding stages (He et al., 2026; NS and Li, 2024). Furthermore, relatively few investigations combine Pareto-guided policy selection with multi-critic actor–critic learning while simultaneously considering trajectory smoothness, productivity, and industrial safety constraints. Finally, comprehensive validation that integrates simulation experiments, quantitative benchmarking, vision-guided localization, and real automotive spot-welding deployment remains scarce. These limitations motivate the development of the proposed Pareto-Guided Multi-Objective DDPG framework, which unifies adaptive multi-objective reinforcement learning with industrial trajectory planning and real-world validation for autonomous cobot spot welding.

## 3 Problem definition and industrial system

The autonomous spot-welding problem is formulated by considering the industrial manufacturing environment, robotic system constraints, and reinforcement learning framework within a unified optimization model. The proposed formulation captures the interactions between the collaborative robot, welding targets, workspace geometry, and operational limitations to accurately represent practical automotive production scenarios. Furthermore, the trajectory planning task is modeled as a multi-objective Markov decision process that simultaneously optimizes positioning accuracy, motion smoothness, operational safety, and manufacturing productivity.

### 3.1 Overall system framework

The proposed Pareto-Guided Multi-Objective Deep Deterministic Policy Gradient (PMO-DDPG) framework provides an integrated closed-loop architecture for autonomous trajectory planning and execution in automotive spot welding. The system combines computer-aided process planning, industrial perception, reinforcement learning, motion generation, collision-aware control, and execution monitoring within a unified optimization framework. Unlike conventional robotic programming approaches that rely on manually generated trajectories, the proposed framework continuously optimizes robot motion according to multiple operational objectives while incorporating feedback from the industrial environment. The overall workflow consists of eight sequential yet interconnected stages that exchange information through a continuous feedback mechanism, allowing the reinforcement learning agent to refine future decisions according to execution performance.

The workflow begins with the CAD vehicle model and welding plan, where the body-in-white (BIW) model, welding-point coordinates, and manufacturing specifications are extracted from the digital product definition. The environment perception and localization module subsequently identifies the current welding target using industrial vision sensors while estimating robot pose, obstacle locations, and workspace geometry. These observations are transformed into a compact state representation containing robot joint states, end-effector pose, target information, environmental perception, welding force, and process phase. The resulting state vector is supplied to the PMO-DDPG agent, where an actor network generates continuous control actions and four objective-specific critics simultaneously evaluate trajectory accuracy, motion smoothness, operational safety, and productivity. A Pareto preference module dynamically adjusts objective importance before updating the policy through deterministic policy gradients. The generated Cartesian trajectory is then validated by the trajectory planning module and verified through constraint handling, where joint limits, velocity limits, acceleration constraints, collision avoidance, and welding-force restrictions are enforced before execution. After autonomous spot welding is completed, performance indicators including positioning accuracy, motion smoothness, cycle time, energy consumption, collision events, and welding quality are recorded and transferred to the feedback module. This closed-loop learning process enables continuous policy improvement across consecutive welding tasks.

Figure 1 presents the overall workflow of the proposed PMO-DDPG framework for autonomous cobot trajectory planning in automotive spot welding, illustrating both the online execution pipeline and the reinforcement learning training process. The workflow begins with the CAD vehicle model and welding plan, where predefined welding-point coordinates, target orientations, manufacturing constraints, and robot parameters are extracted and forwarded to the perception module. Environment perception and localization integrate robot pose estimation, RGB-D sensing, point-cloud processing, obstacle detection, welding-force estimation, and localization confidence to construct a comprehensive description of the current manufacturing scene. These measurements are transformed into the Markov state representation, which combines robot kinematics, end-effector pose, target information, obstacle distances, estimated welding force, and the current welding phase. The resulting state vector is processed by the PMO-DDPG agent, where the actor network generates continuous motion commands while four objective-specific critics independently evaluate trajectory accuracy, motion smoothness, operational safety, and production efficiency. The Pareto preference module dynamically aggregates these objectives using adaptive phase-dependent weights and maintains a non-dominated policy archive to guide policy optimization. The optimized actions are subsequently converted into collision-free robot trajectories that satisfy joint, velocity, workspace, and safety constraints before being executed by the industrial cobot. During execution, robot telemetry, process measurements, and welding outcomes are continuously collected and returned to both the environment model and the performance evaluation module through dedicated feedback pathways. In parallel, replay-buffer sampling, critic updates, target-network updates, and Pareto-based policy refinement are performed through the training loop, while closed-loop performance feedback enables continuous adaptation across successive welding points. The explicit representation of forward information flow, actor–critic interactions, reinforcement learning updates, and execution feedback demonstrates how perception, decision-making, control, and learning are seamlessly integrated into a unified framework for robust industrial robotic spot welding.

**FIGURE 1:**
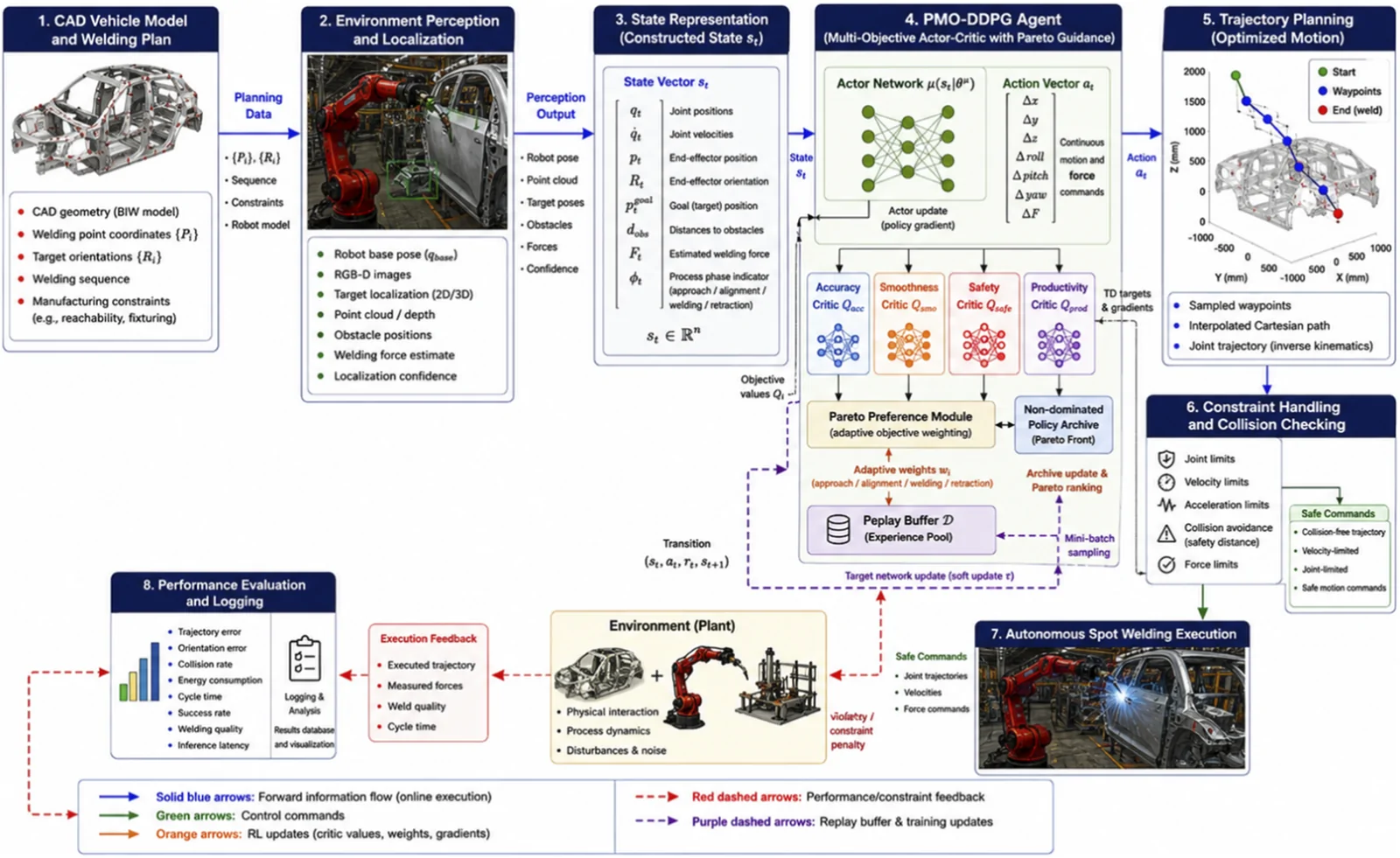
Overall framework of the proposed PMO-DDPG spot-welding system. Solid blue arrows denote online forward information flow, black internal arrows denote actor-critic computations, and dashed arrows denote training or inter-weld feedback.

Mathematically, the complete framework can be represented as Equation 1

$$s_{t+1}=f\left(s_{t},\pi \left(s_{t}\right),E\right),$$ (1)

where $s_{t}$ denotes the current system state, $\pi \left(\cdot \right)$ represents the learned PMO-DDPG policy, and E denotes the industrial spot-welding environment. The objective of the learning framework is to determine an optimal policy (Equation 2)

$$\pi^{*}=\arg\max_{\pi }E\left[\sum_{t=0}^{T}\gamma^{t}r_{t}\right],$$ (2)

where $r_{t}$ is the aggregated multi-objective reward and γ is the discount factor.

### 3.2 Automotive spot-welding environment

The industrial environment considered in this work models a typical automotive body-in-white spot-welding workstation equipped with a six-degree-of-freedom collaborative manipulator carrying a resistance spot-welding gun. The robot operates within a structured manufacturing cell containing fixed workholding fixtures, vehicle-body assemblies, safety fences, and predefined welding locations. Unlike simplified benchmark environments, the industrial workspace contains narrow passages, metallic structures, and multiple collision hazards that require accurate trajectory generation and continuous safety monitoring. Consequently, the reinforcement learning agent must simultaneously optimize trajectory quality, productivity, and operational safety while respecting physical robot limitations.

Figure 2 illustrates the industrial automotive spot-welding workstation considered in this study, including the collaborative robot, body-in-white structure, resistance spot-welding gun, workholding fixtures, predefined welding points, safety-restricted regions, robot reach envelope, and global workspace coordinate frame. The vehicle body contains a sequence of predefined welding points represented by the set (Equation 3)

$$W=\left\{w_{1},w_{2},\ldots ,w_{N}\right\},$$ (3)

where N denotes the total number of weld locations. Each welding point is associated with a desired Cartesian position

$$p_{i}={\left[x_{i},y_{i},z_{i}\right]}^{T}$$ (4)

and orientation (Equations 4, 5)

$$R_{i}\in SO\left(3\right),$$ (5)

which define the required pose of the welding gun. During execution, the robot sequentially visits these target locations while minimizing unnecessary motion between consecutive welds. Workholding fixtures rigidly secure the vehicle body throughout the welding process, ensuring positional consistency and reducing structural vibration. Safety-restricted regions define prohibited volumes around obstacles and surrounding equipment, while the robot reach envelope limits the feasible operating space according to manipulator kinematics. All robot motions are represented in the global workspace coordinate frame (Equation 6)

$$F_{B}=\left\{X,Y,Z\right\},$$ (6)

which serves as the reference coordinate system for localization, trajectory generation, and industrial execution.

**FIGURE 2:**
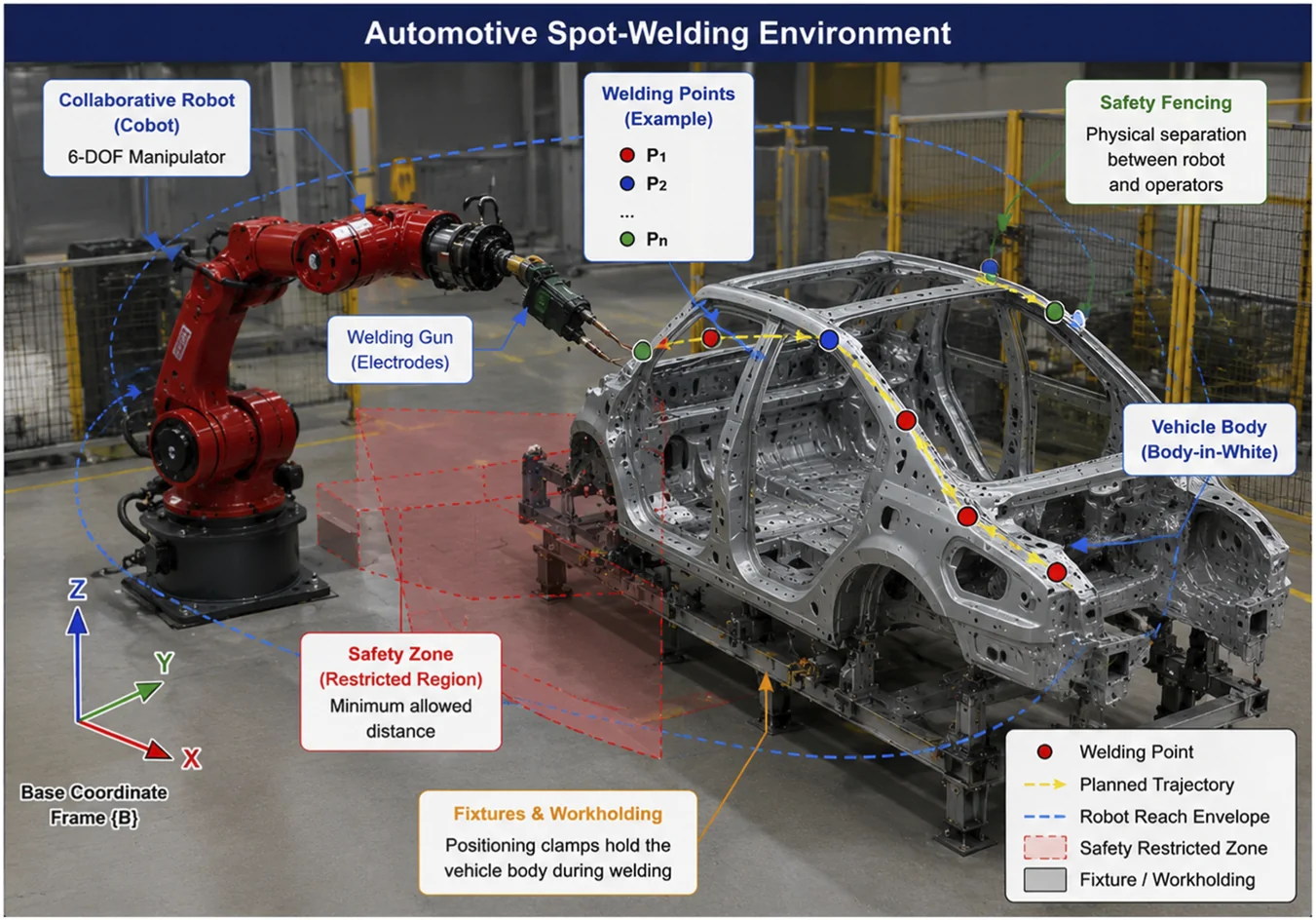
Automotive spot-welding environment with collaborative robot.

### 3.3 Markov decision process formulation

The autonomous trajectory planning problem is formulated as a Markov Decision Process (MDP), as defined in Equation 7. This formulation establishes the mathematical foundation of the reinforcement learning framework by describing the interaction between the collaborative robot and the industrial environment in terms of states, actions, state transitions, reward functions, and long-term optimization objectives.

$$M=\left(S,A,P,R,\gamma \right),$$ (7)

where S denotes the state space, A represents the continuous action space, P is the state-transition probability, R denotes the reward function, and $\gamma \in \left[0,1\right]$ is the discount factor.

$$M=\left(S,A,P,R,\gamma \right),$$ (8)

The system state at time t is defined as Equation 9

$$s_{t}=\left[q_{t},{\dot{q}}_{t},p_{t},R_{t},p_{t}^{\;w},d_{t},F_{t},\phi_{t},ID_{t}\right],$$ (9)

where $q_{t}$ and ${\dot{q}}_{t}$ denote robot joint positions and velocities, $p_{t}$ and $R_{t}$ represent end-effector position and orientation, $p_{t}^{\;w}$ is the target welding pose, $d_{t}$ contains obstacle-distance measurements obtained from the perception system, $F_{t}$ denotes the welding force, $\phi_{t}$ identifies the current welding phase (approach, alignment, welding, or retraction), and $ID_{t}$ specifies the active welding-point index.

The continuous action generated by the actor network is expressed as Equation 10

$$a_{t}=\left[\Delta x,\Delta y,\Delta z,\Delta \alpha ,\Delta \beta ,\Delta \gamma ,\Delta F\right],$$ (10)

where the first six variables correspond to incremental Cartesian translation and orientation updates, while ΔF adjusts the commanded welding force. After applying the selected action, the industrial environment determines the subsequent state according to Equation 11

$$s_{t+1}∼P\left(s_{t+1}|s_{t},a_{t}\right),$$ (11)

which incorporates robot dynamics, sensor feedback, obstacle interactions, and welding process responses.

### 3.4 Operational constraints

Safe industrial deployment requires the planned trajectory to satisfy multiple physical and operational constraints throughout the welding cycle. Joint positions must remain within the mechanical limits of the collaborative robot (Equation 12),

$$q_{i}^{\min}\leq q_{i}\leq q_{i}^{\max},$$ (12)

while joint velocities and accelerations satisfy (Equation 13)

$$|{\dot{q}}_{i}|\leq {\dot{q}}_{i}^{\max},|{\ddot{q}}_{i}|\leq {\ddot{q}}_{i}^{\max}.$$ (13)

Collision avoidance is enforced by maintaining a minimum safety distance (Equation 14)

$$d_{\text{obs}}\geq d_{\text{safe}},$$ (14)

between the robot and surrounding obstacles. Furthermore, all end-effector positions must remain inside the reachable workspace, p∈Ω, where Ω denotes the allowable workspace volume. Welding force is constrained according to Equation 15

$$F_{\min}\leq F_{t}\leq F_{\max},$$ (15)

to ensure reliable weld formation while preventing electrode damage. Finally, production efficiency requires each welding cycle to satisfy $T_{\text{cycle}}\leq T_{\max},$ thereby maintaining compatibility with automotive manufacturing throughput requirements.

### 3.5 Multi-objective optimization problem

Trajectory planning is formulated as a multi-objective optimization problem that simultaneously balances positioning accuracy, motion smoothness, operational safety, and production efficiency. The objective vector associated with a policy π is defined as Equation 16a

$$F\left(\pi \right)=\left[F_{acc},F_{smo}{,F}_{safe}{,F}_{prod}\right]$$ (16a)

These four high-level objectives are evaluated by four independent critic networks within the PMO-DDPG architecture. Internally, the critics aggregate six lower-level reward components corresponding to trajectory accuracy, orientation precision, motion smoothness, collision avoidance, energy efficiency, and productivity. Rather than employing fixed scalar weights, the proposed framework adaptively adjusts the contribution of each objective according to the current welding phase using Pareto-guided preference weighting. Consequently, the learned policy seeks non-dominated solutions that achieve accurate positioning, smooth robot motion, safe obstacle avoidance, and high production efficiency simultaneously, making the framework well suited for autonomous industrial spot-welding applications.

## 4 Materials and methods

The proposed Pareto-Guided Multi-Objective Deep Deterministic Policy Gradient (PMO-DDPG) framework integrates multi-objective reinforcement learning with adaptive Pareto optimization to generate safe, accurate, and efficient trajectories for autonomous collaborative robotic spot welding. The methodological framework encompasses the overall system architecture, actor–critic network design, multi-objective reward formulation, phase-dependent preference weighting, Pareto-guided policy optimization, training strategy, and computational complexity analysis.

### 4.1 Proposed PMO-DDPG architecture

The proposed Pareto-Guided Multi-Objective Deep Deterministic Policy Gradient (PMO-DDPG) framework extends the conventional DDPG algorithm by introducing multiple objective-specific critic networks together with an adaptive Pareto-guided preference module for autonomous trajectory planning in automotive spot welding. Unlike traditional actor-critic reinforcement learning methods that optimize a single scalar objective, the proposed framework decomposes the optimization process into several physically meaningful objectives that are evaluated independently before being adaptively aggregated. This architecture enables simultaneous optimization of trajectory accuracy, motion smoothness, operational safety, and production efficiency while preserving stable learning characteristics.

As illustrated in Figure 3, the proposed architecture consists of six major computational modules, namely, the state encoder, actor network, industrial environment, objective-specific critic networks, Pareto preference module, and replay memory with target networks. At every decision instant, the robot observes the system state (Equation 16b)

$$s_{t}=\left[q_{t},{\dot{q}}_{t},p_{t},R_{t},P_{t}^{w},R_{t}^{w},{ID}_{t},d_{t}^{obs},F_{t},\emptyset_{t}\right],$$ (16b)

where.

1.
$q_{t}$ denotes robot joint positions,
2.
${\dot{q}}_{t}$ represents joint velocities,
3.
$p_{t}$ and $R_{t}$ denote the current end-effector position and orientation,
4.
$p_{t}^{w}$ and $R_{t}^{w}$ correspond to the desired welding pose,
5.
$ID_{t}$ identifies the active welding point,
6.
$d_{t}^{obs}$ represents obstacle-distance measurements,
7.
$F_{t}$ denotes the welding force, and
8.
$\phi_{t}$ specifies the current welding phase.

**FIGURE 3:**
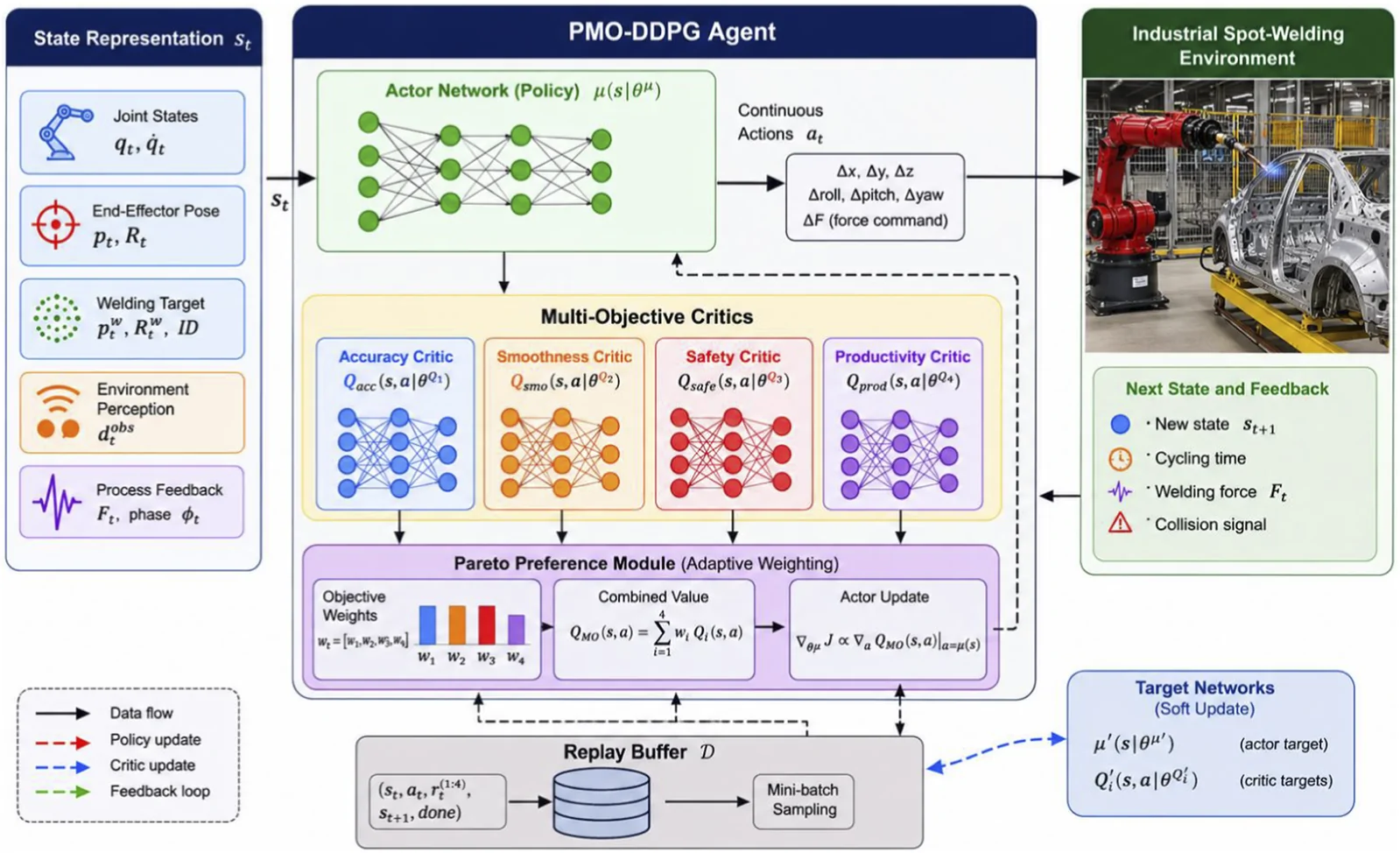
Architecture of the proposed PMO-DDPG framework.

The encoded state is forwarded to the actor network, which generates continuous control commands for Cartesian motion and force regulation. These actions are executed within the industrial welding environment, producing the next state together with objective-specific rewards. Four independent critic networks estimate the long-term value associated with each optimization objective, whereas the Pareto preference module dynamically adjusts their relative importance according to the current welding phase. The replay buffer stores transition tuples for off-policy learning, while slowly updated target actor and target critics stabilize optimization by reducing oscillations during temporal-difference learning.

The interaction between the learning agent and the industrial environment follows (Equation 17):

$$s_{t+1}=f\left(s_{t},a_{t}\right),$$ (17)

where $f\left(\cdot \right)$ represents the robot dynamics, welding process, sensor feedback, and environmental interactions.

### 4.2 Actor network

The actor network is responsible for generating continuous robot actions that directly control manipulator motion during spot welding. Since industrial robots operate in continuous Cartesian space, deterministic policy learning is considerably more suitable than discrete action selection methods. The actor approximates the policy

$$\mu \left(s|\theta^{\mu }\right),$$ (18)

where $\theta^{\mu }$ denotes the learnable parameters of the policy network.

The network receives the state vector defined in Equation 16b and processes it using multiple fully connected hidden layers with nonlinear activation functions. Batch normalization is employed to improve numerical stability by reducing internal covariate shift, while Rectified Linear Unit (ReLU) activation promotes efficient gradient propagation during optimization. The output layer employs the hyperbolic tangent activation function (Equation 19)

$$\hat{a}=\tanh\left(z\right),$$ (19)

which constrains every output dimension to the interval $-1\leq {\hat{a}}_{i}\leq 1.$ These normalized outputs are subsequently transformed into physically feasible robot commands according to Equation 20

$$a_{i}=a_{i}^{\min}+\frac{{\hat{a}}_{i}+1}{2}\left(a_{i}^{\max}-a_{i}^{\min}\right),$$ (20)

where $a_{i}^{\min}$ and $a_{i}^{\max}$ denote the mechanical limits of the corresponding actuator.

The resulting continuous action vector is expressed as Equation 21

$$a_{t}=\left[\Delta x,\Delta y,\Delta z,\Delta \alpha ,\Delta \beta ,\Delta \gamma ,\Delta F\right],$$ (21)

where the first six components represent incremental Cartesian translations and rotations, whereas ΔF adjusts the commanded welding force.

During training, exploration is achieved by adding Gaussian noise to the deterministic policy Equation 22

$$a_{t}=\mu \left(s_{t}\right)+N_{t},$$ (22)

where $N_{t}∼N\left(0,\sigma^{2}\right).$

This exploration strategy enables the robot to discover alternative trajectories while avoiding premature convergence to locally optimal solutions. During deployment, the exploration term is removed, allowing deterministic execution of optimized trajectories suitable for industrial applications.

### 4.3 Objective-specific critic networks

A major limitation of conventional DDPG is that a single critic attempts to approximate multiple conflicting objectives using one scalar value function. In industrial spot welding, however, positioning accuracy, motion smoothness, collision avoidance, and production efficiency exhibit different optimization characteristics and frequently conflict with one another. Consequently, compressing these objectives into a single critic often leads to unstable learning and suboptimal policies.

To overcome this limitation, the proposed PMO-DDPG framework introduces four independent critic networks, $Q_{acc}\left(s,a\right)$ , $Q_{smo}\left(s,a\right)$ , $Q_{safe}\left(s,a\right)$ , and $Q_{prod}\left(s,a\right)$ , corresponding respectively to trajectory accuracy, motion smoothness, operational safety, and manufacturing productivity.

Each critic estimates the expected discounted return associated with its individual objective (Equation 23),

$$Q_{k}\left(s_{t},a_{t}\right)=E\left[\sum_{i=t}^{\infty }\gamma^{i-t}r_{i}^{k}\right]$$ (23)

where $k\in \left\{acc,smo,safe,prod\right\}.$

The target value for each critic is computed independently using the corresponding target critic (Equation 24),

$$y_{t}^{k}=r_{t}^{k}+\gamma Q_{k}^{\prime }\left(s_{t+1},\mu^{\prime }\left(s_{t+1}\right)\right)$$ (24)

where $Q_{k}^{\prime }$ and $\mu^{\prime }$ denote the target critic and target actor networks, respectively.

The critic parameters are optimized by minimizing the temporal-difference loss (Equation 25),

$$L_{k}=\frac{1}{B}\sum_{i=1}^{B}{\left(Q_{k}\left(s_{i},a_{i}\right)-y_{i}^{k}\right)}^{2}$$ (25)

where B denotes the mini-batch size sampled from the replay buffer.

Compared with a conventional single-critic architecture, objective decomposition offers several advantages. First, each critic specializes in learning the dynamics of one performance criterion, thereby reducing approximation interference among competing objectives. Second, the independent critics provide richer optimization information that can be adaptively weighted according to the current welding phase. Third, the modular design improves training stability because inaccuracies in one critic do not directly distort the value estimates of unrelated objectives. Finally, the multi-critic representation naturally supports Pareto-based policy optimization, enabling the actor to balance accuracy, safety, smoothness, and productivity without relying on manually selected fixed reward coefficients.

The outputs of these four critics are subsequently integrated by the adaptive Pareto preference module described in the following subsection, allowing the policy to dynamically shift its optimization priorities throughout different stages of the spot-welding process.

### 4.4 Multi-objective reward mechanism

Unlike conventional reinforcement learning methods that optimize a single scalar reward, the proposed PMO-DDPG framework evaluates multiple performance objectives independently before combining them through adaptive Pareto-guided weighting (Park et al., 2026). This design enables the learning agent to distinguish between trajectory tracking quality, motion smoothness, operational safety, and manufacturing productivity rather than forcing all optimization criteria into a single reward signal. Consequently, the proposed reward mechanism produces more informative learning feedback and reduces conflicts among competing objectives during policy optimization. The complete reward generation process is illustrated in Figure 4, where six physically meaningful reward components are first computed and subsequently grouped into four high-level optimization objectives.

**FIGURE 4:**
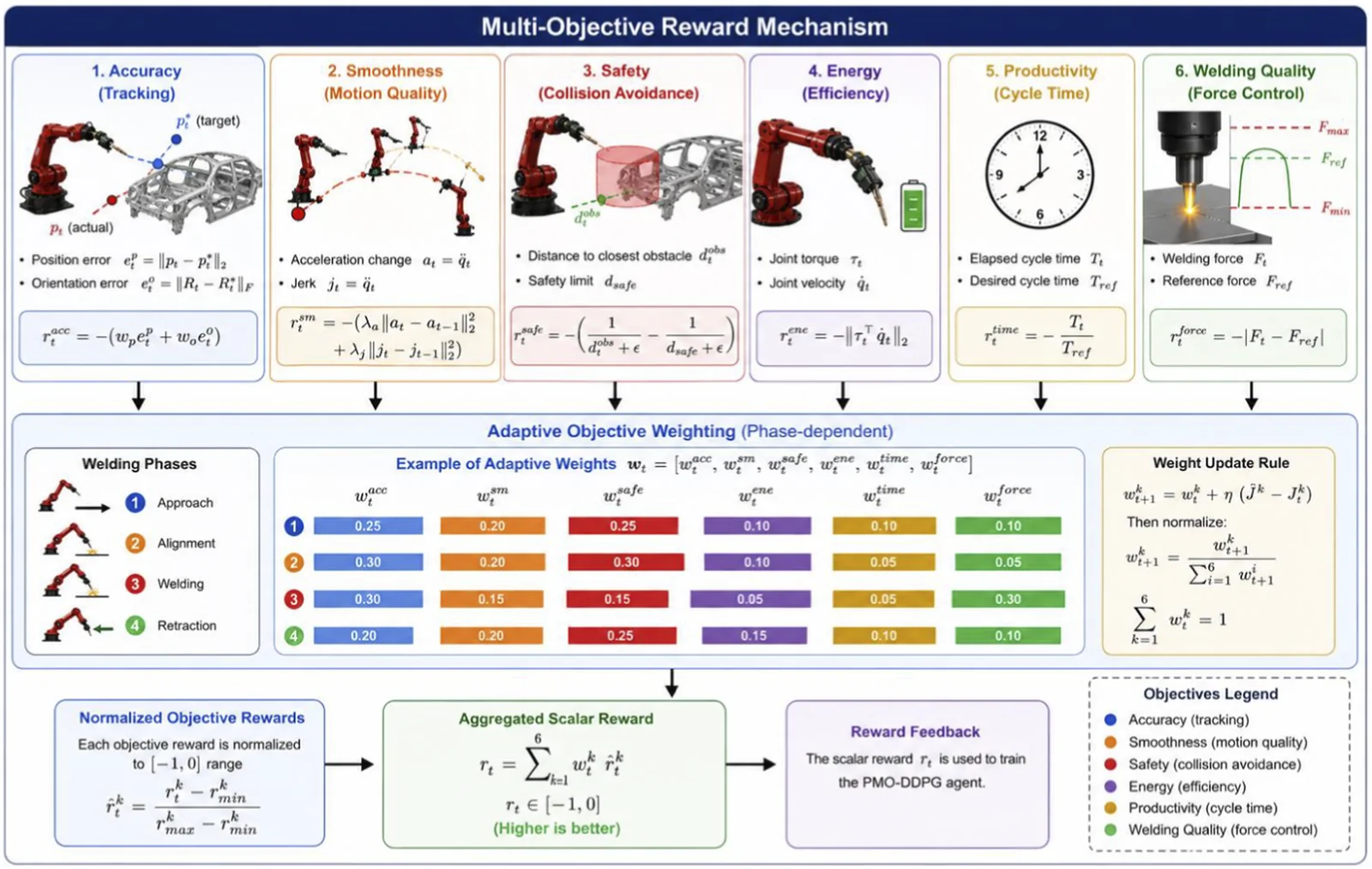
Multi-objective reward mechanism with adaptive weighting.

The first reward component evaluates trajectory tracking accuracy by jointly considering the position and orientation errors of the welding gun. The position error is defined as

$$e_{t}^{p}={∥p_{t}-p_{t}^{*}∥}_{2}$$ (26)

where $p_{t}$ denotes the current end-effector position and ${p*}_{t}$ represents the desired welding pose. Likewise, the orientation error is computed as

$$e_{t}^{o}={∥R_{t}-R_{t}^{*}∥}_{F}$$ (27)

where $R_{t}$ and ${R*}_{t}$ denote the current and target orientation matrices, respectively. The corresponding tracking reward is expressed as

$$\begin{array}{ll} r_{t}^{position} & =-e_{t}^{p}\\ & \;r_{t}^{orientation}=-e_{t}^{o} \end{array}$$ (28)

The second objective evaluates trajectory smoothness. Excessive acceleration or jerk increases mechanical wear, vibration, and electrode instability during welding. Therefore, acceleration and jerk penalties are introduced as

$$\begin{array}{ll} r_{t}^{acceleration} & =-{∥{\ddot{q}}_{t}∥}_{2}^{2},\\ & \;r_{t}^{jerk}=-{∥{q...}_{t}∥}_{2}^{2}. \end{array}$$ (29)

These penalties encourage continuous robot motion while eliminating abrupt velocity variations.

The third objective represents operational safety. Safe robot motion requires maintaining sufficient clearance from surrounding obstacles while completely avoiding collisions. Let $d_{t}^{obs}$ denote the minimum obstacle distance measured by the perception system. The safety-distance reward is formulated as

$$r_{t}^{distance}=-\frac{1}{d_{t}^{obs}+\varepsilon }$$ (30)

where ε prevents numerical instability when the robot approaches nearby obstacles. Collision events receive an additional penalty

$$r_{t}^{collision}=\left\{\begin{array}{cc} -1, & \text{collision}\\ 0, & \text{otherwise}. \end{array}\right.$$ (31)

The fourth objective measures mechanical energy consumption. Since industrial production requires energy-efficient robot motion, actuator torque and joint velocity are jointly considered,

$$r_{t}^{energy}=-|\tau_{t}^{T}{\dot{q}}_{t}|$$ (32)

where $\tau_{t}$ represents joint torque.

The fifth objective evaluates productivity through cycle-time minimization,

$$r_{t}^{time}=-\frac{T_{t}}{T_{ref}}$$ (33)

where $T_{t}$ denotes the elapsed execution time and $T_{ref}$ is the desired production cycle.

Finally, accurate force regulation is essential for obtaining consistent weld quality. The force-tracking reward is defined as

$$r_{t}^{force}=-|F_{t}-F_{ref}|$$ (34)

where $F_{t}$ is the measured electrode force and $F_{ref}$ is the desired welding force.

Instead of optimizing these six components independently, they are grouped into four high-level critic objectives,

$$r_{t}^{acc}=r_{t}^{position}+r_{t}^{orientation}+r_{t}^{force},$$ (35)

$$r_{t}^{smo}=r_{t}^{acceleration}+r_{t}^{jerk},$$ (36)

$$r_{t}^{safe}=r_{t}^{distance}+r_{t}^{collision},$$ (37)

$$r_{t}^{prod}=r_{t}^{energy}+r_{t}^{time}.$$ (38)

The six terms above are component rewards rather than six alternative overall reward functions. Each component is normalized to [-1, 0] using the same training-set bounds, with values closer to zero indicating better performance. The final scalar reward used for actor optimization is the six-term phase-weighted combination in Equation 53. For critic learning, the components are grouped into four returns: the accuracy critic receives tracking and force components, the smoothness critic receives acceleration and jerk, the safety critic receives clearance and collision information, and the productivity critic receives energy and time components. Thus, Equations 26–38 define interpretable component signals and critic targets, whereas Equation 53 is the final overall optimization reward.

### 4.5 Phase-dependent adaptive preference weighting

Industrial spot welding consists of several operational stages that impose different control priorities on the robotic manipulator. For example, during the approach phase the robot primarily focuses on collision avoidance and smooth motion, whereas precise positioning and force regulation become substantially more important immediately before welding (Liu et al., 2026). Consequently, assigning constant objective weights throughout the entire welding cycle cannot adequately represent the varying requirements of industrial production.

The proposed PMO-DDPG framework therefore introduces the six-component phase-dependent preference vector

$$\beta_{t}=\left[\beta_{t}^{\text{acc}},\beta_{t}^{\text{sm}},\beta_{t}^{\text{safe}},\beta_{t}^{\text{ene}},\beta_{t}^{\text{time}},\beta_{t}^{\text{force}}\right]$$ (39)

subject to Equation 40

$$\sum_{k=1}^{6}\beta_{t}^{k}=1,\beta_{t}^{k}\geq 0$$ (40)

The preference weights vary according to the current welding phase (Equation 41)

$$\phi_{t}\in \left\{Approach,Alignment,Welding,Retraction\right\}.$$ (41)

The base phase-dependent weights used in the experiments are given in Table 1. To make the final reward specification explicit, the vectors are $\beta_{Approach}$ = (0.25, 0.20, 0.25, 0.10, 0.10, 0.10), $\beta_{Alignment}$ = (0.30, 0.20, 0.30, 0.10, 0.05, 0.05), $\beta_{Welding}$ = (0.30, 0.15, 0.15, 0.05, 0.05, 0.30), and $\beta_{Retraction}$ = (0.20, 0.20, 0.25, 0.15, 0.10, 0.10), ordered as (accuracy, smoothness, safety, energy, time, force). Each vector sums to 1.0 and is used as the phase-specific base before the archive-deficit update and renormalization described below.

**TABLE 1:** Phase-dependent adaptive objective weights used during the four spot-welding stages.

Phase	Accuracy	Smoothness	Safety	Energy	Time	Force
Approach	0.25	0.20	0.25	0.10	0.10	0.10
Alignment	0.30	0.20	0.30	0.10	0.05	0.05
Welding	0.30	0.15	0.15	0.05	0.05	0.30
Retraction	0.20	0.20	0.25	0.15	0.10	0.10

During the approach phase, the manipulator moves from its previous weld toward the next target. Since robot velocity is relatively high, safety and trajectory smoothness receive the highest weighting in order to minimize collision risk and avoid abrupt robot motion.

The alignment phase begins when the welding gun approaches the target location. At this stage, positioning accuracy becomes the dominant objective because even small pose errors may lead to defective weld placement. Safety continues to receive a relatively high weight owing to the close proximity between the electrode and the vehicle body.

During the welding phase, the robot remains nearly stationary while maintaining accurate electrode positioning and constant welding force. Consequently, the preference vector allocates the largest weight to trajectory accuracy, including position, orientation, and force-tracking rewards, whereas productivity temporarily receives lower importance because maintaining weld quality is the primary manufacturing objective.

Finally, during the retraction phase, the electrode withdraws from the completed welding point before moving toward the next target. At this stage, motion smoothness and productivity become the principal optimization objectives because the manipulator must leave the confined workspace efficiently while minimizing unnecessary acceleration and energy consumption.

The tabulated vector is the phase-specific base preference. It is refined using the performance-deficit rule shown in Figure 4, ${\tilde{\beta }}_{t+1}^{k}=\max\;\left(0,\beta_{t}^{k}+\eta \left({\overset{ˉ}{J}}^{k}-J_{t}^{k}\right)\right)$ , followed by $\beta_{t+1}^{k}=\frac{{\tilde{\beta }}_{t+1}^{k}}{\sum_{i=1}^{6}{\tilde{\beta }}_{t+1}^{i}}$ . Here, $J_{t}^{k}$ is the current normalized objective score and ${\overset{ˉ}{J}}^{k}$ is the corresponding reference score derived from the non-dominated archive. The six component preferences are then mapped to four critic weights as $w_{\text{acc}}=\beta_{\text{acc}}+\beta_{\text{force}},w_{\text{smo}}=\beta_{\text{sm}},w_{\text{safe}}=\beta_{\text{safe}},w_{\text{prod}}=\beta_{\text{ene}}+\beta_{\text{time}}$ . This mapping preserves the four-critic architecture while making the final reward and the influence of every physical component explicit.

### 4.6 Pareto-guided preference update and critic aggregation

Pareto guidance operates at two connected levels. At scheduled evaluation checkpoints, the current policy is evaluated with the four trajectory-level objectives and inserted into an archive only if it is non-dominated; previously stored policies that become dominated are removed. The archive ideal point supplies the reference objective values ${\overset{ˉ}{J}}^{k}$ used by the adaptive preference update above. These updated preferences are mapped to the four critic weights and therefore change the aggregated action-value function and the actor gradient in Equations 42–45. After training, Pareto dominance is applied again to the complete candidate set, and Equation 57 selects the non-dominated policy closest to the requested industrial operating preference. Pareto dominance is not differentiated directly; it affects learning through the reference-driven preference weights and affects deployment through non-dominated policy retention and selection.

The aggregated multi-objective action-value function is defined as

$$Q_{MO}\left(s,a\right)=\sum_{k=1}^{4}w_{t}^{k}Q_{k}\left(s,a\right),$$ (42)

where $Q_{k}\left(s,a\right)$ denotes the output of the corresponding objective-specific critic and $w_{t}^{k}$ represents the adaptive phase-dependent preference weight defined in Equation 39.

The actor parameters are optimized by maximizing the aggregated critic,

$$J\left(\theta^{\mu }\right)=\frac{1}{N}\sum_{i=1}^{N}Q_{MO}\left(s_{i},\mu \left(s_{i}\right)\right),$$ (43)

where N denotes the mini-batch size sampled from the replay buffer.

Applying the deterministic policy gradient theorem yields

$$\nabla_{\theta^{\mu }}J=\frac{1}{N}\sum_{i=1}^{N}\nabla_{a}Q_{MO}\left(s_{i},a\right){|}_{a=\mu \left(s_{i}\right)}\nabla_{\theta^{\mu }}\mu \left(s_{i}\right),$$ (44)

which can be further expanded as

$$\nabla_{a}Q_{MO}=\sum_{k=1}^{4}w_{t}^{k}\nabla_{a}Q_{k}$$ (45)

Equation 45 shows the direct effect on deep reinforcement learning: each critic contributes an independent action gradient, and the Pareto-informed phase weight multiplies that gradient before the actor parameters are updated. A deficient safety objective therefore increases the safety critic’s contribution, while a policy already near the archive ideal for energy receives less energy-driven pressure. This prevents one objective from silently dominating solely because of reward scale.

Following critic and actor optimization, the target networks are updated using soft parameter updates (Equation 46),

$$\theta^{\prime }\leftarrow \tau \theta +\left(1-\tau \right)\theta^{\prime },$$ (46)

where τ≪1 denotes the soft-update coefficient. This gradual parameter synchronization significantly improves training stability by reducing oscillations in temporal-difference learning.

Compared with conventional DDPG, the proposed Pareto-guided aggregation strategy provides three important advantages. First, conflicting objectives remain explicitly separated throughout learning, preventing interference between unrelated optimization criteria. Second, adaptive weighting enables the optimization priorities to evolve naturally across different welding phases without manual reward redesign. Third, the resulting policy converges toward Pareto-efficient solutions that simultaneously improve trajectory accuracy, motion smoothness, operational safety, and production efficiency, making the proposed framework particularly suitable for complex industrial spot-welding applications.

### 4.7 PMO-DDPG training procedure

The proposed PMO-DDPG framework is trained using an off-policy actor-critic strategy that integrates objective-specific critics with adaptive Pareto-guided optimization. Compared with conventional DDPG, the proposed learning procedure simultaneously estimates four independent value functions while maintaining a single deterministic actor responsible for generating continuous robot actions. This architecture enables the policy to learn balanced control strategies that satisfy multiple industrial objectives without introducing significant computational complexity during online execution.

Figure 5 illustrates the overall training workflow of the proposed framework. Training begins with initialization of the actor network, four objective-specific critics, target networks, replay buffer, and adaptive preference module. At the beginning of every episode, the industrial spot-welding environment is reset, including the robot configuration, active welding-point sequence, obstacle information, and process variables. The current system state is subsequently encoded according to Equation 16b and provided to the actor network, which generates a continuous control action. The selected action is executed within the industrial environment, producing the subsequent robot state together with multiple objective-specific rewards. These transitions are stored in the replay buffer and randomly sampled during optimization, thereby reducing temporal correlation among consecutive experiences and improving learning stability.

**FIGURE 5:**
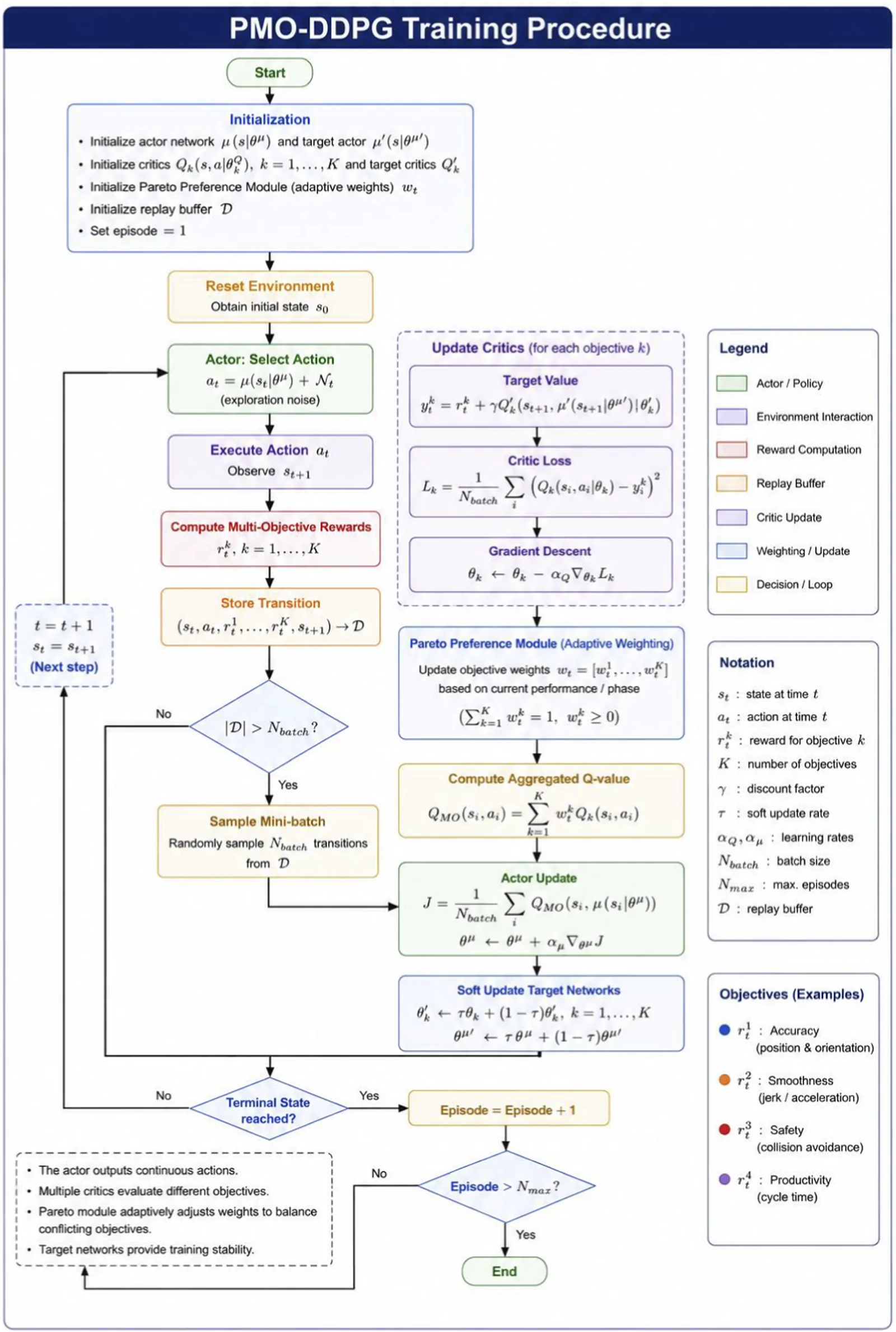
PMO-DDPG training procedure.

The interaction between the learning agent and the environment is represented by the transition tuple (Equations 47, 48)

$$T_{t}=\left(s_{t},a_{t},r_{t},s_{t+1},d_{t}\right),$$ (47)

where

$$r_{t}=\left[r_{t}^{acc},r_{t}^{smo},r_{t}^{safe},r_{t}^{prod}\right],$$ (48)

contains the four objective-specific rewards and $d_{t}$ denotes the terminal-state indicator.

The replay memory stores a collection of transitions (Equation 49)

$$D=\left\{T_{1},T_{2},\ldots ,T_{M}\right\},$$ (49)

where M denotes the replay-buffer capacity. During every optimization iteration, a mini-batch of transitions is randomly sampled from D , ensuring approximately independent observations and improving convergence of the stochastic gradient updates.

For each sampled transition, the target value of every critic is computed independently using its corresponding target network (Equation 50),

$$y_{i}^{k}=r_{i}^{k}+\gamma \left(1-d_{i}\right)Q_{k}^{\prime }\left(s_{i+1},\mu^{\prime }\left(s_{i+1}\right)\right),$$ (50)

where $k\in \left\{acc,smo,safe,prod\right\}.$

Each critic is subsequently optimized by minimizing the temporal-difference error defined in Equation 25. After all critics have been updated, their outputs are combined using the adaptive Pareto preference weights described in Section 4.6, allowing the actor network to receive a unified optimization signal while preserving objective-specific learning.

The actor parameters are then updated using the deterministic policy gradient,

$$\theta^{\mu }\leftarrow \theta^{\mu }+\eta_{\mu }\nabla_{\theta^{\mu }}J,$$ (51)

where $\eta_{\mu }$ denotes the actor learning rate. Finally, the target actor and target critics are softly synchronized according to Equation 46, reducing abrupt parameter variations and improving optimization stability throughout training.

Listing 1 Pareto-guided multi-objective deep deterministic policy gradient (PMO-DDPG) training algorithm.

**Input**: Environment E ; actor network $\mu \left(s|\theta^{\mu }\right)$ ; objective-specific critics $Q_{k}\left(s,a|\theta^{Q_{k}}\right)$ , k=1,…,K ; target networks; replay buffer D ; batch size B ; discount factor γ ; soft-update coefficient τ ; maximum episodes $N_{\text{ep}}$

**Output**: Trained PMO-DDPG policy $\mu *\left(s\right)$

1. Initialize the actor network, objective-specific critic networks, target networks, replay buffer, and pareto preference vector
2. for each episode $=1,\ldots ,N_{\text{ep}}$ do
3.  Reset the automotive spot-welding environment and observe the initial state $s_{0}$

4.  while the episode is not terminated do
5.   Generate the continuous action $a_{t}=\mu \left(s_{t}|\theta^{\mu }\right)+N_{t}.$

6.   Execute the action, observe $s_{t+1}$ , and compute the objective rewards $r_{t}^{\text{acc}},r_{t}^{\text{smo}},r_{t}^{\text{safe}},r_{t}^{\text{prod}}.$

7.   Compute the adaptive aggregated reward $r_{t}=\sum_{k=1}^{K}w_{t}^{k}r_{t}^{k}.$

8.   Store the transition $\left(s_{t},a_{t},\left\{r_{t}^{k}\right\},s_{t+1},d_{t}\right)$

in the replay buffer
9.   Sample a mini-batch from D

10.   for each critic $Q_{k}$ do
11.    Compute the target value $y_{i}^{k}=r_{i}^{k}+\gamma \left(1‐d_{i}\right)Q_{k}^{\prime }\left(s_{i+1},\mu^{\prime }\left(s_{i+1}\right)\right).$

12.    Update the critic by minimizing $L_{k}=\frac{1}{B}\sum_{i=1}^{B}{\left(Q_{k}\left(s_{i},a_{i}\right)-y_{i}^{k}\right)}^{2}.$

13.   end for
14.   Combine all critic outputs using pareto-guided weighting and update the actor through the deterministic policy gradient.
15.   Soft-update the actor and critic target networks using coefficient τ

16.   Set $s_{t}\leftarrow s_{t+1}$

17.  end while
18. end for
19. Evaluate the trained policies and select the non-dominated solution from the pareto set.
20. Return the optimized policy $\mu *\left(s\right)$

The complete optimization procedure is summarized in Listing 1, which presents the entire PMO-DDPG training algorithm from network initialization to final policy generation. As shown in the listing, the learning process consists of four principal stages: interaction with the industrial environment, acquisition of objective-specific rewards, replay-buffer learning with critic optimization, and actor update through Pareto-guided critic aggregation. Unlike conventional DDPG, the proposed algorithm concludes by evaluating the learned candidate policies and selecting the non-dominated solution from the Pareto set, ensuring that the final controller provides a balanced compromise among accuracy, smoothness, safety, and productivity rather than maximizing only a single objective.

### 4.8 Adaptive reward evaluation and Pareto policy selection

Although multiple objective-specific rewards provide richer learning information than a single scalar reward, a practical industrial controller ultimately requires one executable policy. The proposed framework therefore introduces an adaptive reward evaluation strategy followed by Pareto-guided policy selection to identify the most appropriate operating policy for the current manufacturing requirements.

Initially, the six normalized component rewards described in Section 4.4 are computed independently. The adaptive preference module then selects and updates the six-component weight vector in Equation 52 according to the current phase and Pareto-reference deficit.

$$\beta_{t}=\left[\beta_{t}^{acc},\beta_{t}^{sm},\beta_{t}^{safe},\beta_{t}^{ene},\beta_{t}^{time},\beta_{t}^{force}\right]$$ (52)

Using these adaptive weights, the final scalar optimization reward is

$$r_{t}=\beta_{t}^{acc}{\hat{r}}_{t}^{acc}+\beta_{t}^{sm}{\hat{r}}_{t}^{sm}+\beta_{t}^{safe}{\hat{r}}_{t}^{safe}+\beta_{t}^{ene}{\hat{r}}_{t}^{ene}+\beta_{t}^{time}{\hat{r}}_{t}^{time}+\beta_{t}^{force}{\hat{r}}_{t}^{force}$$ (53)

Equation 53, not any individual component reward, is the overall reward used for actor optimization. The six component values remain available for critic grouping, diagnostic analysis, and Pareto evaluation.

After training, every candidate policy is evaluated over multiple spot-welding episodes. The resulting trajectory-level performance is summarized by the objective vector (Equation 54)

$$F\left(\pi_{i}\right)=\left[F_{acc},F_{smo},F_{safe},F_{prod}\right],$$ (54)

Instead of ranking policies according to a single weighted objective, Pareto dominance is used to compare competing solutions. A policy $\pi_{i}$ dominates another policy $\pi_{j}$ when

$$F_{k}\left(\pi_{i}\right)\leq F_{k}\left(\pi_{j}\right),\forall k,$$ (55)

and $F_{k}\left(\pi_{i}\right)<F_{k}\left(\pi_{j}\right)$ for at least one objective (Equation 55).

The set of non-dominated policies is therefore expressed as Equation 56

$$P^{*}=\left\{\pi_{i}\in \Pi :∄\pi_{j}\text{ dominating }\pi_{i}\right\}$$ (56)

where Π denotes the complete candidate-policy set.

When several Pareto-optimal policies exist, the preferred operating solution is selected by minimizing the weighted Euclidean distance to the ideal objective vector,

$$\pi^{*}=\arg\min_{\pi_{i}\in P^{*}}\sqrt{\sum_{k=1}^{4}\rho_{k}{\left({\hat{F}}_{k}-F_{k}^{ideal}\right)}^{2}},$$ (57)

where $\rho_{k}$ represents the decision-maker preference coefficients. This final selection procedure enables the industrial operator to choose operating points that emphasize productivity, safety, or trajectory quality according to practical production requirements without retraining the reinforcement learning model.

Listing 2 Adaptive multi-objective reward evaluation and Pareto-guided policy selection.

**Input: state** $s_{t}$ **; action** $a_{t}$ **; next state** $s_{t+1}$ **; welding phase** $\phi_{t}$ **; target pose; obstacle distances; force; motion variables; candidate-policy archive A.**
**Output: final scalar reward** $r_{t}$ ; updated archive A; selected pareto-optimal policy π*
 1. Compute the six physical reward components for accuracy, smoothness, safety, energy, time, and force
 2. Normalize every component to the common interval [−1, 0]
 3. Load the base component-weight vector for the current welding phase from the phase matrix
 4. Update and normalize the component weights using the archive-reference performance deficit
 5. Compute the final scalar reward with Equation 53.

$r_{t}=\beta_{t}^{acc}{\hat{r}}_{t}^{acc}+\beta_{t}^{sm}{\hat{r}}_{t}^{sm}+\beta_{t}^{safe}{\hat{r}}_{t}^{safe}+\beta_{t}^{ene}{\hat{r}}_{t}^{ene}+\beta_{t}^{time}{\hat{r}}_{t}^{time}+\beta_{t}^{force}{\hat{r}}_{t}^{force}$

 6. Map the six component weights to the four critic weights and update the objective-specific critics
 7. Evaluate candidate policy checkpoints, retain non-dominated policies, and remove dominated archive members
 8. Update the actor using the weighted critic gradient in Equations 42–45
           
$Q_{MO}\left(s,a\right)=\sum_{k=1}^{4}w_{t}^{k}Q_{k}\left(s,a\right),$

           
$J\left(\theta^{\mu }\right)=\frac{1}{N}\sum_{i=1}^{N}Q_{MO}\left(s_{i},\mu \left(s_{i}\right)\right),$

        
$\nabla_{\theta^{\mu }}J=\frac{1}{N}\sum_{i=1}^{N}\nabla_{a}Q_{MO}\left(s_{i},a\right){|}_{a=\mu \left(s_{i}\right)}\nabla_{\theta^{\mu }}\mu \left(s_{i}\right),$

            
$\nabla_{a}Q_{MO}=\sum_{k=1}^{4}w_{t}^{k}\nabla_{a}Q_{k}$

 9. After training, select the policy closest to the ideal point using Equation 57
   
$\pi *=\arg\min_{\pi_{i}\in P*}\sqrt{\sum_{k=1}^{4}\rho_{k}{\left({\hat{F}}_{k}-F_{k}^{ideal}\right)}^{2}},$

and return π*.

The complete adaptive reward evaluation and Pareto policy-selection procedure is summarized in Listing 2, which details objective calculation, adaptive weight determination, reward aggregation, Pareto dominance evaluation, construction of the non-dominated policy set, and final policy selection.

#### 4.8.1 Computational complexity

The computational complexity of the proposed PMO-DDPG framework is primarily determined by forward inference through the actor network and the four objective-specific critic networks. Let $L_{\mu }$ denote the number of parameters of the actor network and $L_{Q}$ represent the number of parameters in a single critic. During online deployment, the computational complexity of policy inference is $O\left(L_{\mu }\right)$ , since only the actor network is required to generate continuous robot actions. Consequently, the online execution complexity remains comparable to that of conventional DDPG and satisfies the real-time requirements of industrial robot control.

During training, the four critics are evaluated independently, yielding an overall complexity of $O\left(L_{\mu }+4L_{Q}\right)$ , for each optimization iteration. Although this increases computational cost compared with a single-critic DDPG framework, the critic updates are performed offline during learning and therefore do not affect real-time deployment.

The adaptive Pareto preference module introduces only simple vector normalization and weighted summation operations, whose computational complexity is $O\left(K\right)$ , where K=4 denotes the number of optimization objectives. Because K is fixed and small, the computational overhead introduced by adaptive preference weighting is negligible compared with neural-network inference.

Similarly, Pareto policy selection is executed only after completion of training and therefore does not influence online trajectory generation. Consequently, the proposed PMO-DDPG framework preserves the computational efficiency of deterministic actor-based control while significantly improving optimization quality through multi-objective critic decomposition and adaptive Pareto-guided learning. These characteristics make the proposed framework suitable for real-time autonomous trajectory planning in industrial automotive spot-welding applications, where rapid decision-making and reliable execution are equally important.

## 5 Experimental setup

The proposed PMO-DDPG framework was comprehensively evaluated through simulation and real industrial experiments to assess its effectiveness for autonomous cobot trajectory planning in automotive spot welding. The experimental setup was designed to investigate learning convergence, trajectory quality, operational safety, production efficiency, and real-time performance under representative manufacturing conditions. Furthermore, comparative experiments with state-of-the-art reinforcement learning algorithms and conventional trajectory planning methods were conducted to provide a comprehensive evaluation of the proposed approach.

### 5.1 Simulation environment

The proposed Pareto-Guided Multi-Objective Deep Deterministic Policy Gradient (PMO-DDPG) framework was developed and evaluated using the Webots robotics simulator, which provides realistic rigid-body dynamics, collision detection, and industrial robot modeling suitable for reinforcement learning research. A six-degree-of-freedom collaborative robot equipped with a resistance spot-welding gun was employed to perform sequential welding operations on a body-in-white (BIW) automotive assembly. The simulated workstation includes workholding fixtures, safety-restricted areas, static obstacles, and narrow operating spaces representative of practical automotive manufacturing environments. A total of 20 predefined welding points were distributed over the vehicle body, requiring the robot to generate collision-free trajectories while maintaining precise positioning and appropriate welding-gun orientation. An episode was terminated whenever all welding points were successfully completed, the maximum number of control steps was reached, or a collision occurred with surrounding obstacles or restricted regions. To improve policy robustness and generalization, moderate domain randomization was introduced by perturbing the initial robot configuration, target localization uncertainty, and obstacle placement within predefined safety margins at the beginning of each training episode.

### 5.2 Training configuration

The reinforcement learning model was trained using a carefully selected set of hyperparameters to ensure stable convergence and efficient policy optimization. These parameters define the robot configuration, learning process, network optimization strategy, and exploration behavior employed throughout the training procedure.

Table 2 summarizes the experimental configuration used for training and evaluation. Each learning method was trained for 2,000 episodes with a 500-step cap and evaluated over five independent runs. The shared numerical settings were a batch size of 128, γ = 0.99, Adam optimization, a policy learning rate of 1 × 10^−4^, and a value/critic learning rate of 1 × 10^−3^. The soft-update coefficient τ = 0.005 and the 100,000-transition replay buffer applied only to off-policy methods. For PPO and entropy-adjusted PPO, replay memory and soft target updates were not used; each employed separate fully connected stochastic-policy and state-value networks with ReLU hidden layers, optimized on-policy under the same state/action interface and training budget. The archived experiment record does not identify secondary PPO coefficients as separately tuned study variables; therefore, unverified defaults are not reconstructed and this detail is retained as a reproducibility limitation.

**TABLE 2:** Experimental configuration and hyperparameters.

Parameter	Value
Robot platform	6-DOF collaborative robot
End effector	Resistance spot welding gun
Workspace	1.2 × 1.2 × 1.0 m
Number of welding points	20
Simulation platform	Webots/CoppeliaSim
Control frequency	100 Hz
Maximum episode length	500 steps
Number of episodes	2,000
Replay buffer size	100,000
Batch size	128
Discount factor (γ)	0.99
Soft update coefficient (τ)	0.005
Actor learning rate	1 × 10^−4^
Critic learning rate	1 × 10^−3^
Optimizer	Adam
Exploration noise	Gaussian
Independent training runs	5 per learning method

### 5.3 Comparison methods

The proposed PMO-DDPG framework was compared with eight representative deep reinforcement learning algorithms, including DDPG, TD3, PPO, SAC, DDPG-DG, Fuzzy-TD3, Entropy-adjusted PPO, and Recurrent SAC, to provide a comprehensive evaluation across contemporary continuous-control approaches. DDPG serves as the baseline deterministic actor–critic algorithm, allowing the contribution of the proposed multi-objective architecture to be isolated. TD3 introduces twin critics and delayed policy updates to alleviate value overestimation, while PPO represents a widely adopted on-policy optimization strategy with stable policy updates. SAC employs entropy regularization to improve exploration and robustness in continuous action spaces. DDPG-DG extends the conventional DDPG framework with dynamic guidance mechanisms, Fuzzy-TD3 integrates fuzzy reasoning into twin-delayed policy optimization, Entropy-adjusted PPO incorporates adaptive entropy regulation to balance exploration and exploitation, and Recurrent SAC enhances SAC by exploiting temporal dependencies through recurrent network structures. To ensure a fair comparison, all reinforcement learning methods were trained under the same simulation environment, training budget, hyperparameter settings, and evaluation protocol, as summarized in Table 3. In addition to learning-based methods, conventional trajectory planning algorithms, including Model Predictive Control (MPC), linear interpolation, cubic spline interpolation, B-spline interpolation, Bézier curve planning, and A* graph search, were evaluated to provide geometric and optimization-based references for trajectory quality, smoothness, and constraint satisfaction. Consequently, the experimental comparison assesses the proposed PMO-DDPG framework against both recent state-of-the-art reinforcement learning algorithms and widely adopted industrial trajectory planning techniques under identical operating conditions.

**TABLE 3:** Controlled machine-learning configurations.

Parameter	DDPG	TD3	PPO	SAC	DDPG-DG	Fuzzy-TD3	Entropy-adjusted PPO	Recurrent SAC	PMO-DDPG (proposed)
Training episodes	2,000	2,000	2,000	2,000	2,000	2,000	2,000	2,000	2,000
Maximum steps per episode	500	500	500	500	500	500	500	500	500
Batch size	128	128	128	128	128	128	128	128	128
Discount factor, γ	0.99	0.99	0.99	0.99	0.99	0.99	0.99	0.99	0.99
Policy learning rate	$1\times 10^{-4}$	$1\times 10^{-4}$	$1\times 10^{-4}$	$1\times 10^{-4}$	$1\times 10^{-4}$	$1\times 10^{-4}$	$1\times 10^{-4}$	$1\times 10^{-4}$	$1\times 10^{-4}$
Value/Critic learning rate	$1\times 10^{-3}$	$1\times 10^{-3}$	$1\times 10^{-3}$	$1\times 10^{-3}$	$1\times 10^{-3}$	$1\times 10^{-3}$	$1\times 10^{-3}$	$1\times 10^{-3}$	$1\times 10^{-3}$
Replay buffer size	100,000	100,000	On-policy	100,000	100,000	100,000	On-policy	100,000	100,000
Soft update coefficient, τ	0.005	0.005	N/A	0.005	0.005	0.005	N/A	0.005	0.005
Optimizer	Adam	Adam	Adam	Adam	Adam	Adam	Adam	Adam	Adam
Exploration strategy	Gaussian noise	Gaussian noise	Stochastic policy	Entropy maximization	Gaussian + DG module	Fuzzy-guided exploration	Adaptive entropy	Recurrent stochastic policy	Gaussian + pareto guidance
Network architecture	Actor + 1 critic	Actor + 2 critics	Actor + value MLPs	Actor +2 critics	Actor +1 critic + DG	Actor +2 fuzzy critics	Actor + value MLPs	Recurrent actor + 2 critics	Actor + 4 objective-specific critics
Objective optimization	Single objective	Single objective	Single objective	Single objective	Single objective	Single objective	Single objective	Single objective	Pareto-guided multi-objective optimization
Adaptive objective weighting	No	No	No	No	No	No	No	No	Yes

Comparability scope: every learning-based row in Figure 6 and Table 4 was obtained by re-evaluating a local implementation under the same spot-welding environment, state/action definitions, 2,000-episode training budget, 500-step episode limit, control frequency, termination criteria, reward components, and evaluation protocol. Shared optimizer and learning-rate settings were fixed, whereas algorithm-specific update mechanisms and nonapplicable items, such as replay memory and τ for PPO, were retained as shown in Table 3. Conventional trajectory planners were executed in the same workspace and evaluated with the same physical metrics, but they were not assigned an RL training budget. Consequently, the reported values are controlled within-study descriptive comparisons rather than direct reuse of results from different published platforms.

**FIGURE 6:**
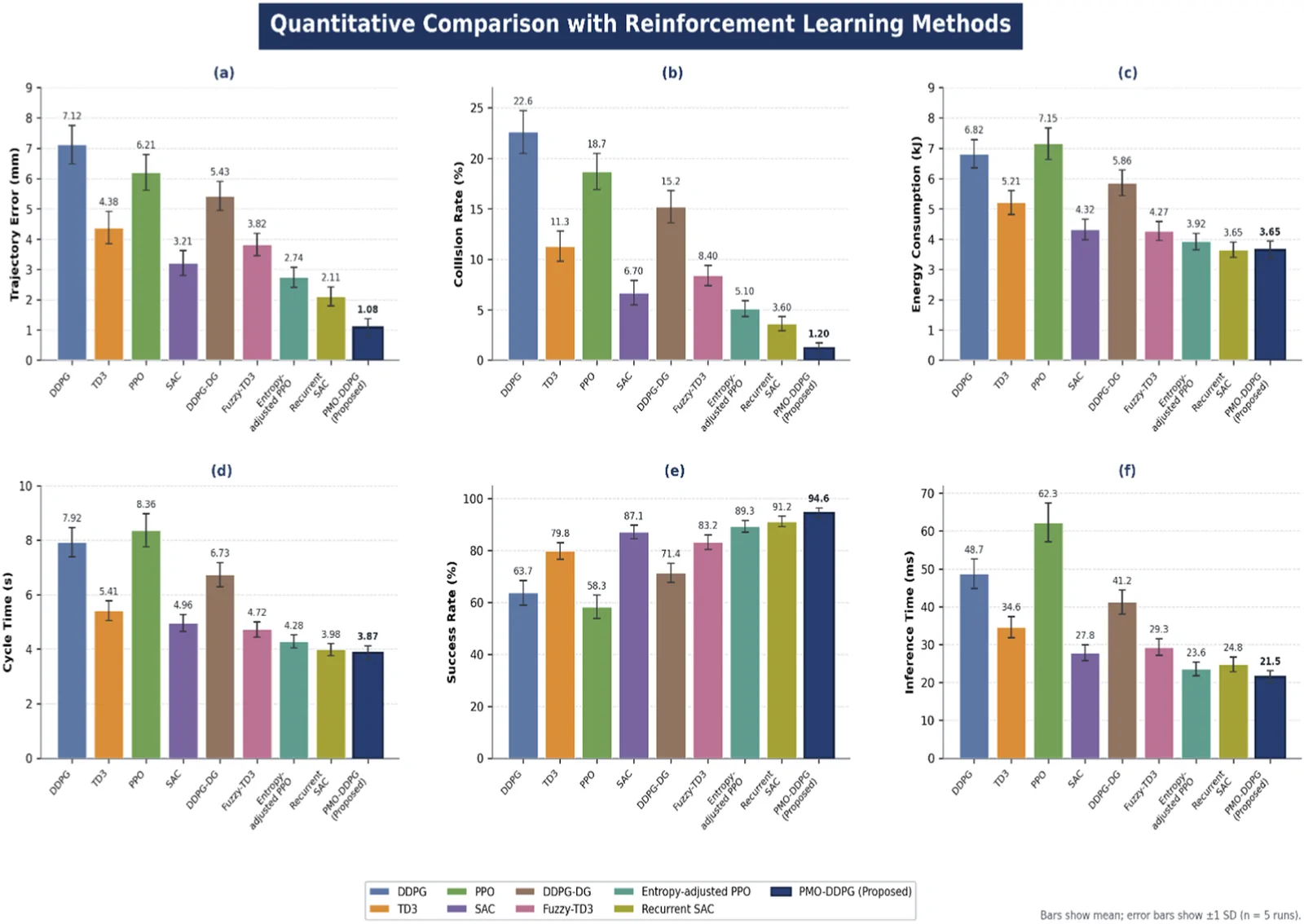
Controlled quantitative comparison of PMO-DDPG with reinforcement learning methods. Bars show the mean and error bars show ±1 standard deviation over five independent runs. **(a)** Trajectory error (mm). **(b)** Collision rate (%). **(c)** Energy consumption (kJ). **(d)** Cycle time (s). **(e)** Success rate (%). **(f)** Inference time (ms).

**TABLE 4:** Controlled within-study quantitative comparison with reinforcement learning methods (Mean ± Standard Deviation, n = 5 Independent Runs).

Method	Trajectory error (mm) ↓	Collision rate (%) ↓	Energy (kJ) ↓	Cycle time (s) ↓	Success rate (%) ↑	Inference time (ms) ↓
DDPG	7.12 ± 0.63	22.6 ± 2.1	6.82 ± 0.47	7.92 ± 0.54	63.7 ± 4.8	48.7 ± 3.9
TD3	4.38 ± 0.54	11.3 ± 1.5	5.21 ± 0.39	5.41 ± 0.36	79.8 ± 3.2	34.6 ± 2.8
PPO	6.21 ± 0.59	18.7 ± 1.8	7.15 ± 0.52	8.36 ± 0.61	58.3 ± 4.5	62.3 ± 5.1
SAC	3.21 ± 0.41	6.7 ± 1.2	4.32 ± 0.34	4.96 ± 0.31	87.1 ± 2.6	27.8 ± 2.1
DDPG-DG	5.43 ± 0.48	15.2 ± 1.6	5.86 ± 0.42	6.73 ± 0.45	71.4 ± 3.7	41.2 ± 3.2
Fuzzy-TD3	3.82 ± 0.37	8.4 ± 1.0	4.27 ± 0.31	4.72 ± 0.28	83.2 ± 2.8	29.3 ± 2.2
Entropy-adjusted PPO	2.74 ± 0.33	5.1 ± 0.8	3.92 ± 0.27	4.28 ± 0.24	89.3 ± 2.3	23.6 ± 1.8
Recurrent SAC	2.11 ± 0.31	3.6 ± 0.7	3.65 ± 0.25	3.98 ± 0.22	91.2 ± 2.0	24.8 ± 1.9
PMO-DDPG (Proposed)	**1.08 ± 0.29**	**1.2 ± 0.5**	**3.65 ± 0.28**	**3.87 ± 0.25**	**94.6 ± 1.8**	**21.5 ± 1.6**

Bold values indicate the best mean performance in each metric among the compared methods. Lower values are better for trajectory error, collision rate, energy consumption, cycle time, and inference time; higher values are better for success rate. Energy consumption is tied at the reported precision (3.65 kJ) for Recurrent SAC and PMO-DDPG. Values are mean ± standard deviation over five independent runs; boldface does not indicate statistical significance.

### 5.4 Experimental rigor and reproducibility

All values in the experiment results are reported as the arithmetic mean ± sample standard deviation across n = 5 independent training runs for each learning method; the Figure 6 error bars represent ±1 standard deviation. The runs used the common 2,000-episode budget, identical 500-step episode limits, consistent state-action interfaces and termination rules, and candidate-policy evaluation without exploration noise. Domain randomization perturbed the initial configuration, target localization, and obstacle placement. Because only five independent runs were available and no formal hypothesis test was conducted, the between-method differences are interpreted descriptively and are not presented as statistically significant.

The simulation study was complemented by n = 100 repeated industrial trials with vision-guided target correction and planned-versus-executed trajectory recording. Repeatability was evaluated from the dispersion of Cartesian and angular errors using the standard-deviation measure in Equation 66, and the full trial traces are presented in Figure 7. These repeated physical tests strengthen the reliability assessment, while confidence intervals and multi-site replication remain subjects for future validation.

**FIGURE 7:**
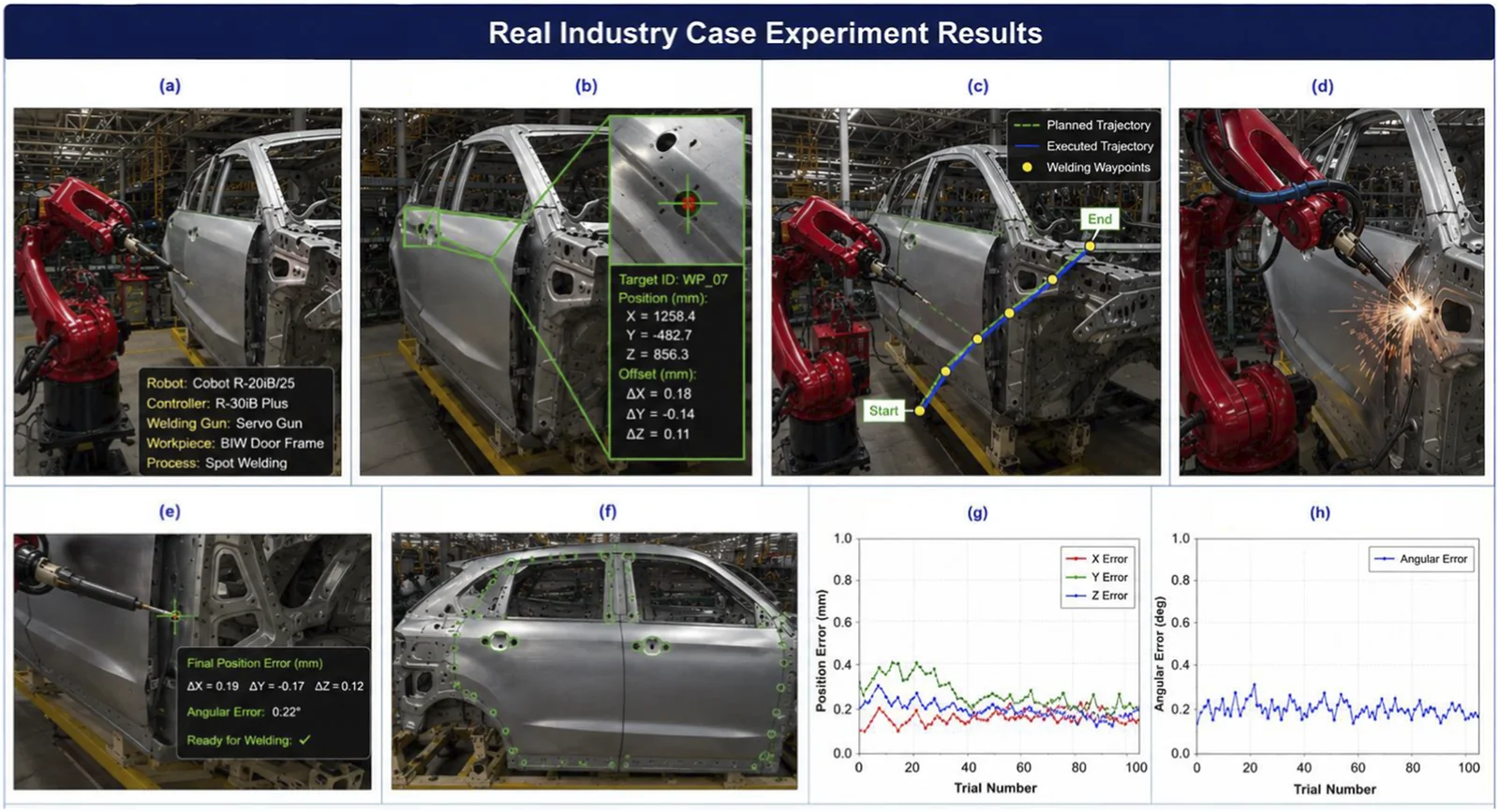
Real industry case experiment results. **(a)** Experimental setup. **(b)** Target localization result. **(c)** Planned vs. Executed trajectory. **(d)** Spot welding in progress. **(e)** Final positioning before welding. **(f)** Completed welds on door frame. **(g)** Positioning error over trials. **(h)** Angular error over trials.

### 5.5 Evaluation metrics

Performance evaluation considered both simulation and industrial criteria to comprehensively assess trajectory quality, operational efficiency, safety, and real-time feasibility. Trajectory accuracy was measured using the Euclidean distance between the generated end-effector position and the desired welding position (Equation 58),

$$e_{\text{traj}}=\frac{1}{N}\sum_{i=1}^{N}{∥p_{i}-p_{i}^{*}∥}_{2},$$ (58)

where $p_{i}$ and ${p*}_{i}$ denote the generated and reference Cartesian positions, respectively. Orientation accuracy was quantified using the Frobenius norm between the desired and estimated rotation matrices (Equation 59),

$$e_{\text{ori}}=\frac{1}{N}\sum_{i=1}^{N}{∥R_{i}-R_{i}^{*}∥}_{F},$$ (59)

Operational safety was evaluated through the collision rate (Equation 60),

$$CR=\frac{N_{\text{collision}}}{N_{\text{episodes}}}\times 100\%,$$ (60)

where $N_{\text{collision}}$ represents the number of episodes containing at least one collision. Energy efficiency was estimated from the cumulative joint mechanical power (Equation 61),

$$E=\sum_{t=1}^{T}|\tau_{t}^{T}{\dot{q}}_{t}|\Delta t,$$ (61)

where $\tau_{t}$ and ${\dot{q}}_{t}$ denote the joint torque and joint velocity vectors, respectively. Production efficiency was measured using the total cycle time (Equation 62),

$$T_{\text{cycle}}=t_{\text{finish}}-t_{\text{start}},$$ (62)

while task completion performance was evaluated by the success rate (Equation 63),

$$SR=\frac{N_{\text{success}}}{N_{\text{episodes}}}\times 100\%,$$ (63)

Real-time applicability was assessed using the average inference time (Equation 64),

$$T_{\inf}=\frac{1}{N}\sum_{i=1}^{N}t_{i}^{\text{forward}},$$ (64)

where $t_{i}^{\text{forward}}$ denotes the forward-pass computation time of the actor network.

Trajectory smoothness was additionally evaluated using the average path curvature (Equation 65),

$$\kappa \left(s\right)=\frac{∥r^{\prime }\left(s\right)\times r^{\prime \prime }\left(s\right)∥}{{∥r^{\prime }\left(s\right)∥}^{3}},$$ (65)

where $r\left(s\right)$ denotes the spatial trajectory parameterized by the path length s . Repeatability was quantified as the standard deviation of the end-effector positions over repeated executions,

$$\sigma_{\text{rep}}=\sqrt{\frac{1}{N-1}\sum_{i=1}^{N}{∥p_{i}-\overset{-}{p}∥}^{2}},$$ (66)

where $\overset{-}{p}$ is the mean trajectory position. During industrial validation, Cartesian positioning accuracy was measured by (Equation 67)

$$e_{\text{pos}}={∥p_{\text{measured}}-p_{\text{desired}}∥}_{2},$$ (67)

whereas angular accuracy was evaluated using the rotational discrepancy between the desired and measured poses (Equation 68),

$$e_{\text{ang}}=\cos^{-1}\left(\frac{\text{trace}\left(R_{\text{desired}}^{T}R_{\text{measured}}\right)-1}{2}\right)$$ (68)

Together, these metrics provide a comprehensive evaluation of trajectory precision, motion smoothness, collision avoidance capability, energy efficiency, computational performance, and practical positioning accuracy under realistic automotive spot-welding conditions.

### 5.6 Real industrial test configuration

Real-world validation was conducted using an automotive spot-welding workstation equipped with a six-degree-of-freedom collaborative robot, a resistance spot-welding gun, and an industrial body-in-white assembly. A vision-based localization system detected welding targets and estimated the offset between the robot tool center point and the desired welding position before trajectory execution. The trajectories generated by PMO-DDPG were executed on the robotic platform while simultaneously recording the planned and actual robot motions. Each experimental scenario was repeated multiple times under identical operating conditions to evaluate positioning accuracy, angular deviation, trajectory consistency, cycle time, and overall robustness, thereby validating the applicability of the proposed framework in practical automotive manufacturing environments.

## 6 Results

The proposed Pareto-Guided Multi-Objective Deep Deterministic Policy Gradient (PMO-DDPG) framework was comprehensively evaluated through simulation and real industrial experiments to assess its capability for autonomous cobot trajectory planning in automotive spot welding. The evaluation considered the convergence characteristics of reinforcement learning, quantitative comparisons with representative reinforcement learning algorithms, multi-objective optimization behavior, trajectory quality, learning evolution, vision-guided localization performance, and real industrial deployment. Both simulation and industrial experiments were analyzed independently to provide an objective assessment of the proposed methodology under controlled and practical manufacturing conditions.

Figure 8 illustrates the learning behavior of the proposed PMO-DDPG framework throughout 2,000 training episodes. The average episode reward increases continuously from approximately −1,000 during the initial exploration stage to nearly 1,000 after convergence, indicating that the reinforcement learning agent progressively discovers more efficient and safer trajectory-planning strategies. Simultaneously, the task success rate increases from below 10% to approximately 93%, demonstrating continuous improvement in reaching designated welding targets while satisfying industrial safety constraints. The actor loss rapidly decreases during early optimization before stabilizing around a constant value, reflecting convergence of the deterministic policy. Likewise, the critic loss decreases from nearly 15 to approximately 1.5, indicating increasingly accurate value estimation for the multiple optimization objectives. Furthermore, the standard-deviation bands surrounding the learning curves gradually narrow during training, confirming reduced policy variance and increasing robustness. These results demonstrate that the proposed multi-objective optimization strategy provides stable reinforcement learning without oscillatory behavior despite simultaneously optimizing accuracy, smoothness, safety, and productivity.

**FIGURE 8:**
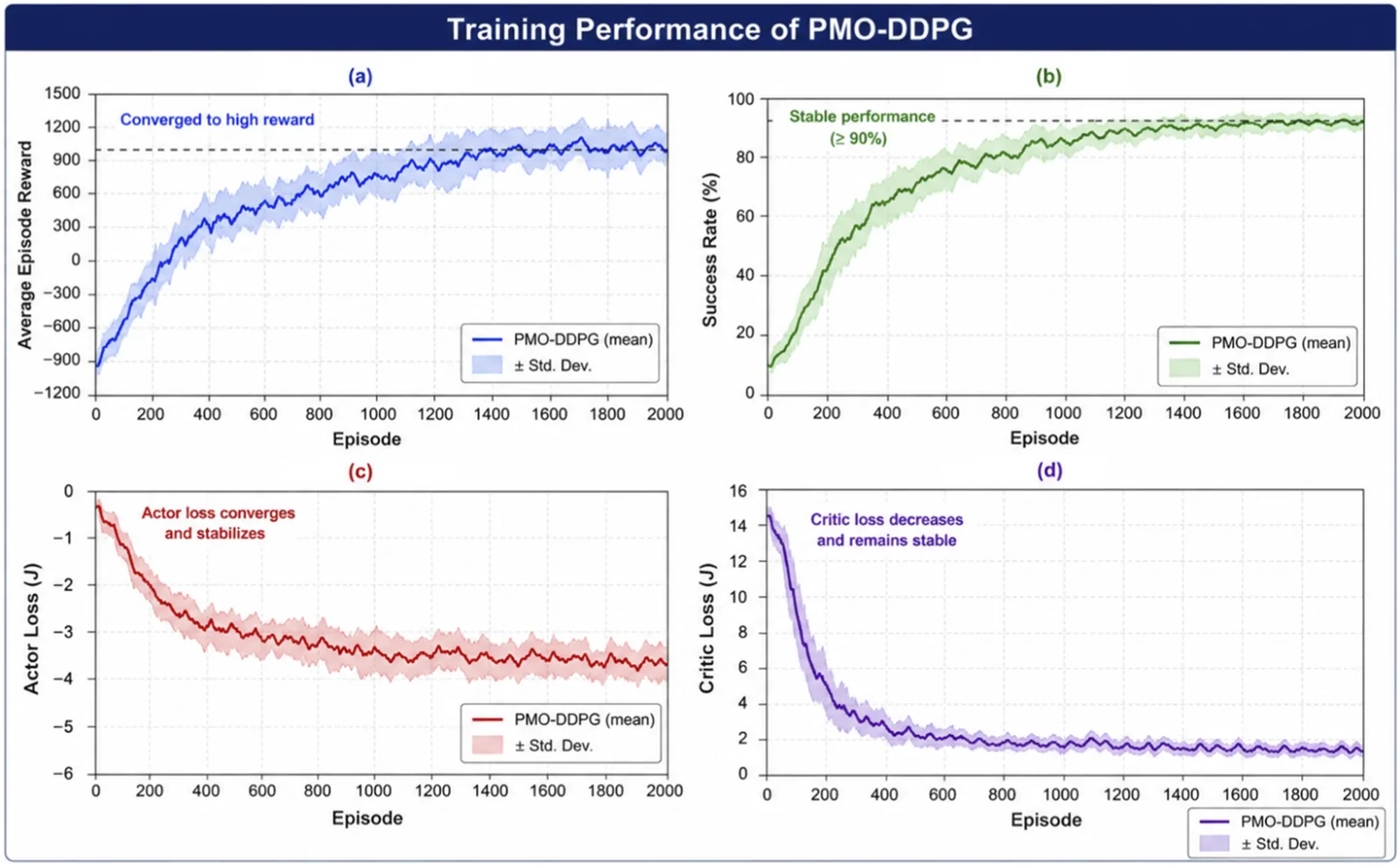
Training performance of the proposed PMO-DDPG. **(a)** Episode reward. **(b)** Success Rate. **(c)** Actor loss. **(d)** Critic loss.

Figure 6 presents the controlled mean ± standard deviation comparison of PMO-DDPG with DDPG, TD3, PPO, SAC, DDPG-DG, Fuzzy-TD3, entropy-adjusted PPO, and recurrent SAC. PMO-DDPG produced the lowest mean trajectory error (1.08 mm), collision rate (1.2%), cycle time (3.87 s), and inference time (21.5 ms), together with the highest mean success rate (94.6%). Its mean energy use was 3.65 kJ, equal to recurrent SAC in Table 4. Relative to the strongest conventional baseline, SAC, the corresponding descriptive reductions were 66.4% in trajectory error, 82.1% in collision rate, 15.5% in energy use, 22.0% in cycle time, and 22.7% in inference latency, while success increased by 7.5 percentage points. These results indicate favorable performance within the shared benchmark. However, because formal significance testing was not performed, they should not be interpreted as proof of universal or statistically significant superiority.

The recent algorithmic variants connect the controlled experiment to current reinforcement-learning developments. Their published performance values were not combined with the present data because the original studies used different robots, tasks, workspaces, and metric definitions. Instead, the plotted rows represent local controlled implementations of the respective algorithmic mechanisms under the shared benchmark described in Sections 5.2–5.4. This design improves within-study comparability but does not establish cross-platform ranking beyond the tested spot-welding scenario.

Table 4 provides the numerical basis for Figure 6. The proposed method has the most favorable mean values for trajectory error, collision rate, cycle time, success rate, and inference time, while its mean energy consumption is tied with recurrent SAC at 3.65 kJ. The reported standard deviations quantify run-to-run variability and are generally smaller for the proposed method than for the conventional baselines. Nevertheless, overlap among some error ranges and the absence of a formal hypothesis test require a restrained interpretation: the table supports a favorable descriptive comparison under the controlled benchmark, not a claim of statistically demonstrated superiority across all robotic platforms or tasks.

Figure 9 illustrates the Pareto front obtained by the proposed PMO-DDPG framework and compares it with DDPG, TD3, PPO, and SAC in terms of the trade-off between trajectory accuracy and energy consumption. All methods exhibit the expected inverse relationship, whereby improved trajectory precision is associated with increased energy expenditure. However, the Pareto curve generated by PMO-DDPG consistently dominates those of the competing algorithms across the entire operating range. For any given level of trajectory accuracy, the proposed framework requires less energy than the baseline methods, while simultaneously achieving higher positioning precision for equivalent energy budgets. This behavior indicates that the adaptive preference weighting mechanism effectively balances conflicting optimization objectives without favoring a single performance criterion. Rather than producing one fixed operating policy, the Pareto-guided optimization generates a set of non-dominated solutions, enabling different operating points to be selected according to production priorities such as maximum precision, minimum energy consumption, or balanced manufacturing efficiency. Compared with conventional scalarized reinforcement learning approaches, PMO-DDPG provides greater flexibility for industrial deployment because decision-makers can adapt the control policy to varying production requirements without retraining the model. Overall, the Pareto-front analysis demonstrates that the proposed multi-objective learning strategy achieves a superior compromise between motion accuracy and energy efficiency, confirming the effectiveness of objective-specific critics and adaptive preference optimization for industrial robotic trajectory planning.

**FIGURE 9:**
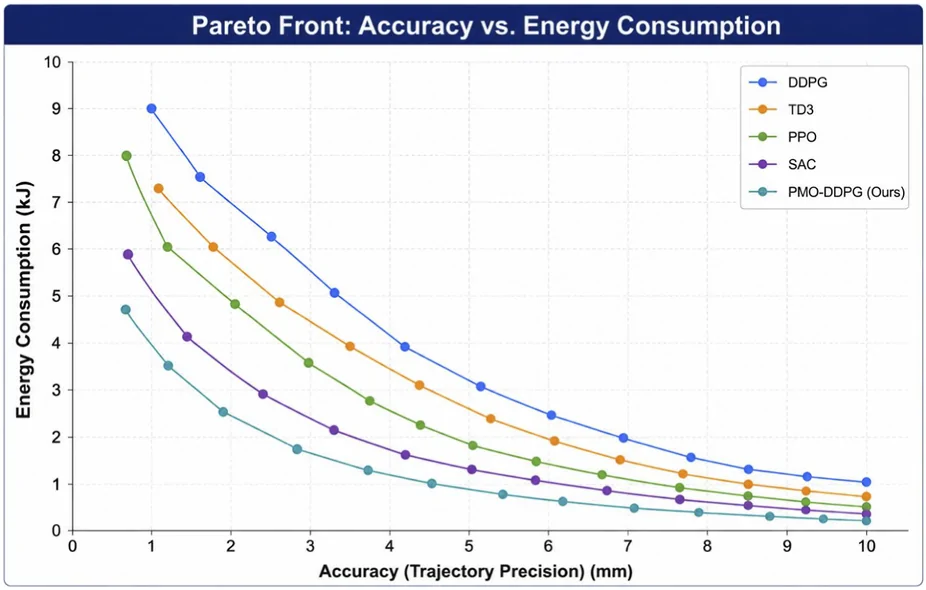
Pareto front analysis of multi-objective optimization.

Figure 10 presents representative simulation results of the proposed PMO-DDPG framework for autonomous trajectory planning in multi-point automotive spot welding. Panel (a) compares the generated trajectories with conventional geometric planners and reinforcement learning methods in a workspace containing multiple welding targets and static obstacles. The proposed PMO-DDPG policy produces the most direct and collision-free trajectories while maintaining smooth transitions between successive welding points. Panels (b) and (c) further demonstrate two representative three-dimensional welding scenarios, where the learned trajectories closely follow the desired motion while exhibiting noticeably smaller deviations than MPC, linear interpolation, cubic spline, and B-spline approaches. In both case studies, PMO-DDPG effectively balances path optimality with obstacle avoidance, resulting in shorter and more efficient motions without abrupt directional changes. Panel (d) quantitatively compares trajectory smoothness through curvature profiles along the entire path. Conventional linear interpolation exhibits the largest curvature fluctuations, whereas cubic spline and B-spline methods reduce but do not eliminate oscillatory behavior. The DDPG baseline generates smoother trajectories than traditional planners; however, the proposed PMO-DDPG consistently maintains the lowest and most stable curvature throughout the motion, indicating superior continuity and dynamic feasibility. These results demonstrate that adaptive Pareto-guided optimization successfully coordinates trajectory accuracy, smoothness, safety, and productivity, enabling the generation of high-quality robotic motions that satisfy the stringent precision and reliability requirements of industrial automotive spot-welding applications.

**FIGURE 10:**
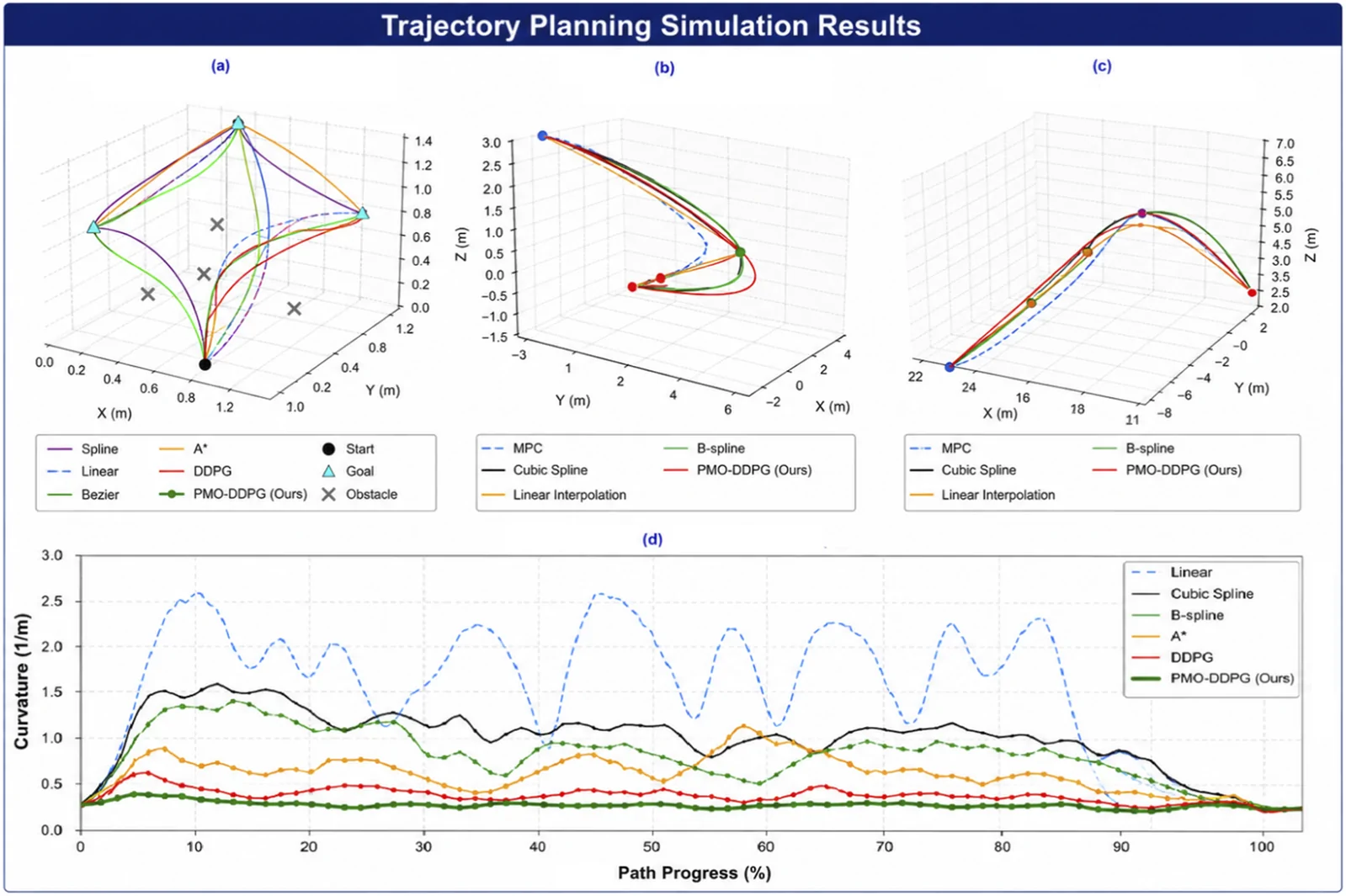
Trajectory planning simulation results. **(a)** Comparision of generated trajectories (Multi-point welding task). **(b)** 3D trajectory Case Study I (x = 0.79, y = 1.05, z = 0.30). **(c)** 3D trajectory Case Study II (x = 17.39, y = −4.16, z = 4.71). **(d)** Trajectory smoothness comparison.

Figure 11 illustrates the progressive evolution of the PMO-DDPG trajectory throughout the reinforcement learning process, demonstrating how the policy gradually improves from exploratory behavior to an optimized motion strategy. During the early training stage (Episode 10), shown in panel (a), the robot explores the workspace extensively, resulting in irregular trajectories with unnecessary detours and inefficient obstacle avoidance. As learning progresses to the intermediate stage (Episode 80) in panel (b), the generated path becomes noticeably smoother, while the robot begins to navigate around obstacles more effectively and follows the desired welding sequence with improved consistency. By the late training stage (Episode 160), illustrated in panel (c), the trajectory closely resembles the reference optimal path, indicating that the learned policy has successfully captured the underlying motion characteristics required for accurate and safe trajectory planning. The final learned policy in panel (d) demonstrates that PMO-DDPG generates a trajectory that is smoother and more direct than conventional planning methods, including linear interpolation, Bézier curves, cubic splines, and the standard DDPG approach. The corresponding trajectory error curve in panel (e) further confirms stable policy convergence, showing a continuous reduction in positioning error from approximately 80 mm during the exploration phase to below 10 mm after convergence. These results demonstrate that the proposed multi-objective reinforcement learning framework effectively balances exploration and exploitation while progressively improving trajectory accuracy, obstacle avoidance capability, and motion smoothness, ultimately producing a robust policy suitable for real-time industrial spot-welding applications.

**FIGURE 11:**
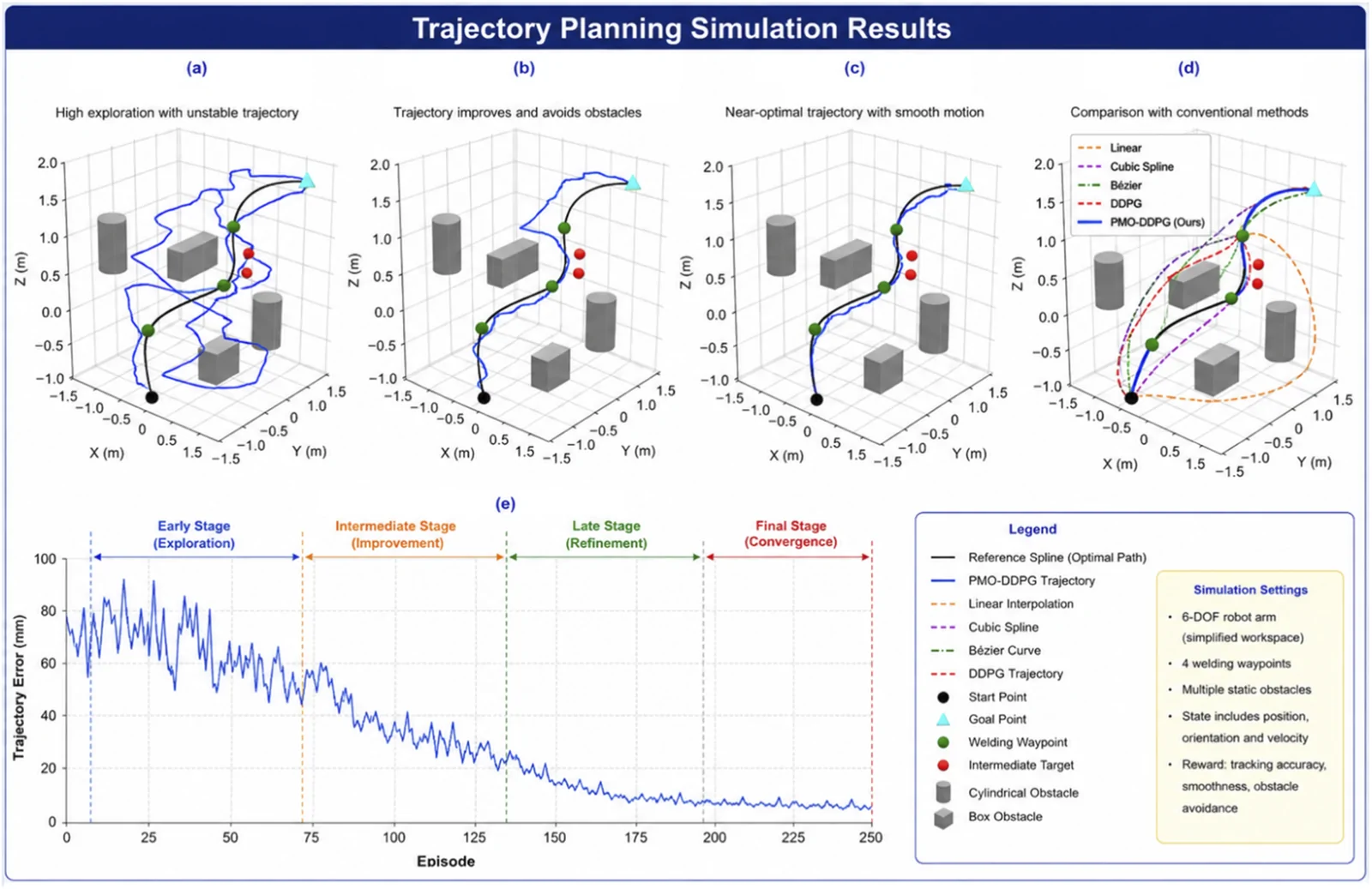
Evolution of the PMO-DDPG trajectory during training. **(a)** Early training stage (Episode 10). **(b)** Intermediate training stage (Episode 80). **(c)** Late training stage (Episode 160). **(d)** Final learned policy (Episode 250). **(e)** Trajectory error during learning.

Figure 12 demonstrates the integration of real-time computer vision with reinforcement learning-based trajectory planning. Before each welding operation, the vision system detects the target welding point together with the current tool-center-point position and estimates the corresponding spatial offset. The calculated displacement is subsequently incorporated into the reinforcement learning state representation, enabling the actor network to generate corrective Cartesian actions before welding execution. The visualization further displays the required motion correction together with the next robot command, allowing accurate alignment between the welding gun and the detected target. This perception–action feedback loop compensates for positioning deviations caused by fixture tolerances or workpiece placement errors and therefore improves the robustness of autonomous welding operations under realistic manufacturing conditions.

**FIGURE 12:**
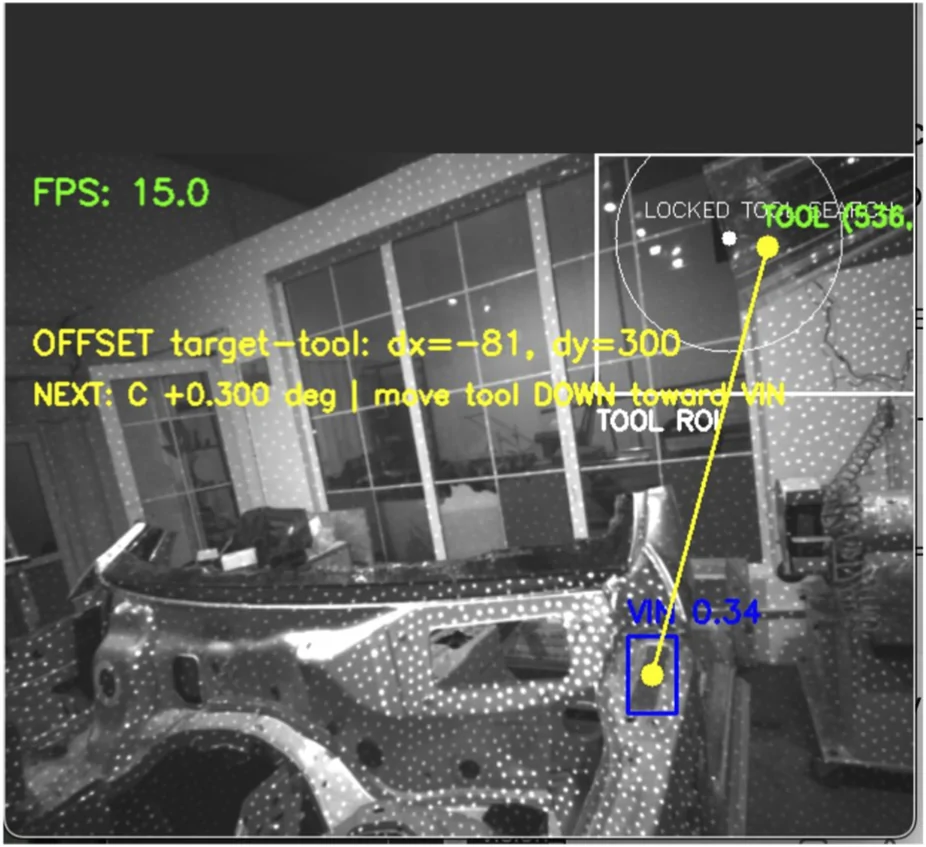
Vision-guided localization and target offset estimation for autonomous spot welding.

Figure 7 presents the real industrial validation of the proposed PMO-DDPG framework in an automotive spot-welding production cell, demonstrating its capability to transfer successfully from simulation to practical deployment. Panel (a) shows the experimental setup consisting of a collaborative robot equipped with a servo resistance welding gun operating on a body-in-white door frame. Panel (b) illustrates the vision-guided localization process, where the target welding point is accurately detected and its spatial offset is estimated with sub-millimeter precision, enabling automatic compensation before trajectory execution. The comparison between the planned and executed trajectories in panel (c) reveals a close correspondence throughout the complete welding sequence, indicating that the learned policy preserves trajectory accuracy under real industrial conditions. Panel (d) demonstrates stable execution of the resistance spot-welding operation, while panel (e) confirms precise positioning immediately before welding, with measured Cartesian errors of ΔX = 0.19 mm, ΔY = −0.17 mm, and ΔZ = 0.12 mm, together with an angular error of only 0.22°, thereby satisfying the accuracy requirements of automotive manufacturing. Panel (f) shows the completed welds distributed consistently across the vehicle door frame, indicating reliable execution of the planned welding sequence. The statistical analyses in panels (g) and (h) further demonstrate excellent repeatability over 100 experimental trials, where the position errors in all three Cartesian directions remain below approximately 0.4 mm and gradually stabilize near 0.2 mm, while the angular error consistently fluctuates around 0.2° with only minor variation. Collectively, these industrial experiments verify that the proposed PMO-DDPG framework provides accurate localization, robust trajectory execution, high repeatability, and reliable real-time performance, confirming its practical suitability for autonomous automotive spot-welding applications.

The experimental evaluation consistently demonstrates the effectiveness of the proposed PMO-DDPG framework for autonomous collaborative robotic spot welding. Stable reinforcement learning convergence was achieved through objective-specific critics and adaptive Pareto-guided optimization, resulting in continuously improving rewards and task success rates. Quantitative comparisons confirmed substantial improvements over representative reinforcement learning algorithms regarding trajectory accuracy, collision avoidance, energy efficiency, production cycle time, and computational performance. The Pareto-front analysis demonstrated the capability of the proposed framework to generate non-dominated operating policies that accommodate different industrial production priorities. Simulation studies further verified smoother trajectories, improved obstacle avoidance, and lower curvature than conventional planning approaches, while the learning evolution analysis illustrated progressive refinement of the trajectory generation policy. Finally, the integration of vision-guided localization with reinforcement learning enabled accurate target detection and highly repeatable industrial spot-welding execution. Collectively, these results confirm that PMO-DDPG provides an efficient, reliable, and practically deployable multi-objective trajectory planning framework for collaborative robotic welding in modern automotive manufacturing.

## 7 Discussion

The experimental results indicate that the proposed Pareto-Guided Multi-Objective Deep Deterministic Policy Gradient (PMO-DDPG) framework addresses several limitations of conventional reinforcement learning for industrial robotic trajectory planning within the tested benchmark. Unlike standard DDPG, the method decomposes the optimization problem into four objective-specific critics for trajectory accuracy, motion smoothness, operational safety, and production efficiency. Phase-dependent preferences then adjust their relative contributions during approach, alignment, welding, and retraction. Across five independent runs, PMO-DDPG exhibited lower mean trajectory error, collision rate, cycle time, and inference latency and higher mean success than the compared methods, with variability reported in Table 4. These are descriptive differences because formal statistical significance was not tested. The observed pattern is consistent with reduced objective interference and phase-aligned learning signals, but broader validation is required before generalizing beyond the present platform and task.

Comparison with state-of-the-art reinforcement learning algorithms further highlights the effectiveness of integrating Pareto-guided optimization into continuous robotic control. Although TD3 and SAC improve learning stability through delayed policy updates and entropy regularization, respectively, both methods still rely on unified value estimation that cannot explicitly distinguish between conflicting industrial objectives. Similarly, PPO demonstrates robust policy optimization but exhibits lower sample efficiency for continuous trajectory planning because of its on-policy learning strategy. In contrast, PMO-DDPG combines the sample efficiency of deterministic actor–critic learning with explicit multi-objective critic decomposition and Pareto-based policy evaluation, enabling the learning agent to identify balanced non-dominated solutions instead of converging toward a single compromise policy. The Pareto-front analysis further demonstrates that the proposed framework offers greater flexibility for industrial deployment by allowing production engineers to select operating points according to different manufacturing priorities, such as maximum positioning accuracy or minimum energy consumption. This capability significantly increases the practical value of reinforcement learning for industrial robotics, where production objectives frequently change according to process requirements and operational constraints.

The successful industrial validation confirms that the proposed framework extends beyond simulation-based optimization and remains effective under realistic automotive manufacturing conditions. The integration of vision-guided target localization with reinforcement learning enables the robot to compensate for positioning uncertainties and workpiece variations through continuous perception–action feedback, resulting in highly repeatable welding operations with sub-millimeter positioning accuracy. Nevertheless, several limitations should be acknowledged. The current study considers a single collaborative robotic platform operating in a static industrial environment with predefined welding sequences and moderate workspace variability. Dynamic obstacles, human–robot collaboration, multi-robot coordination, and online adaptation to changing production schedules were not investigated and remain important directions for future research. In addition, although the proposed framework demonstrates excellent trajectory optimization, future studies could incorporate process-level quality indicators, such as welding current, nugget formation, thermal deformation, and electrode wear, into the multi-objective reinforcement learning framework. Extending the proposed PMO-DDPG architecture with sim-to-real transfer learning, constrained reinforcement learning, and digital-twin technologies may further improve robustness and facilitate deployment in next-generation intelligent automotive manufacturing systems.

The conclusions should be interpreted within the experimental scope. The study uses one cobot, one spot-welding cell, static obstacles, a predefined 20-point welding sequence, moderate rather than unrestricted domain randomization, five independent simulation runs per learning method, and one set of 100 repeated physical trials. Dynamic human motion, multi-robot interference, large production-schedule changes, and long-term electrode degradation were not evaluated. The recent algorithmic variants were compared through controlled local implementations because their published platforms and metrics are not directly commensurate. No formal statistical significance test or multi-site replication was performed. In addition, the measured 21.5 ms mean inference time should not be interpreted as proof of meeting a hard 10 ms deadline implied by 100 Hz control. Public benchmark release, larger multi-site studies, formal inferential testing, dynamic-obstacle experiments, and process-level weld-quality sensing are therefore priorities for future work.

## 8 Conclusion

This paper presented PMO-DDPG for autonomous cobot trajectory planning in automotive spot welding. The method combines four objective-specific critics, six normalized reward components with explicit phase coefficients, phase-dependent preference adaptation, and Pareto-guided policy retention and selection. In the controlled benchmark, PMO-DDPG reduced mean trajectory error by 66.4%, collision rate by 82.1%, energy use by 15.5%, cycle time by 22.0%, and inference latency by 22.7% relative to SAC, while increasing mean success by 7.5 percentage points to 94.6%. The final mean values were 1.08 mm trajectory error, 1.2% collision rate, 3.65 kJ energy use, 3.87 s cycle time, and 21.5 ms inference latency, with standard deviations reported over five independent runs. Because no formal significance test was performed, these outcomes indicate favorable descriptive performance only within the evaluated conditions. The pattern is consistent with specialized critics reducing objective interference, phase weights aligning the learning signal with the current manufacturing stage, and actor-only deployment limiting online computation. Pareto analysis retained multiple non-dominated operating points, and 100 industrial trials demonstrated repeatable vision-guided execution with sub-millimeter positioning. Future work will include inferential testing, multi-site validation, dynamic obstacles, human-robot and multi-robot collaboration, public benchmarks, and process-quality feedback.

## Data Availability

The original contributions presented in the study are included in the article/supplementary material, further inquiries can be directed to the corresponding author.
